# Design and Synthesis of 3-(β-d-Glucopyranosyl)-4-amino/4-guanidino Pyrazole Derivatives and Analysis of Their Glycogen Phosphorylase Inhibitory Potential

**DOI:** 10.3390/molecules28073005

**Published:** 2023-03-28

**Authors:** Sándor Kun, Rachel T. Mathomes, Tibor Docsa, László Somsák, Joseph M. Hayes

**Affiliations:** 1 Department of Organic Chemistry, University of Debrecen, P.O. Box 400, H-4002 Debrecen, Hungary; 2 School of Pharmacy & Biomedical Sciences, University of Central Lancashire, Preston PR1 2HE, UK; 3 Department of Medical Chemistry, Faculty of Medicine, University of Debrecen, 4032 Debrecen, Hungary

**Keywords:** glycogen phosphorylase inhibitor, *C*-glycosyl pyrazole, glucose analogues, ligand strain, MM-GBSA, tautomers, type 2 diabetes

## Abstract

Glycogen phosphorylase (GP) is a key regulator of glucose levels and, with that, an important target for the discovery of novel treatments against type 2 diabetes. β-d-Glucopyranosyl derivatives have provided some of the most potent GP inhibitors discovered to date. In this regard, *C*-β-d-glucopyranosyl azole type inhibitors proved to be particularly effective, with 2- and 4-β-d-glucopyranosyl imidazoles among the most potent designed to date. His377 backbone C=O hydrogen bonding and ion–ion interactions of the protonated imidazole with Asp283 from the 280s loop, stabilizing the inactive state, were proposed as crucial to the observed potencies. Towards further exploring these features, 4-amino-3-(β-d-glucopyranosyl)-5-phenyl-1*H*-pyrazole (**3**) and 3-(β-d-glucopyranosyl)-4-guanidino-5-phenyl-1*H*-pyrazole (**4**) were designed and synthesized with the potential to exploit similar interactions. Binding assay experiments against rabbit muscle GPb revealed **3** as a moderate inhibitor (IC_50_ = 565 µM), but **4** displayed no inhibition at 625 µM concentration. Towards understanding the observed inhibitions, docking and post-docking molecular mechanics—generalized Born surface area (MM-GBSA) binding free energy calculations were performed, together with Monte Carlo and density functional theory (DFT) calculations on the free unbound ligands. The computations revealed that while **3** was predicted to hydrogen bond with His377 C=O in its favoured tautomeric state, the interactions with Asp283 were not direct and there were no ion–ion interactions; for **4**, the most stable tautomer did not have the His377 backbone C=O interaction and while ion–ion interactions and direct hydrogen bonding with Asp283 were predicted, the conformational strain and entropy loss of the ligand in the bound state was significant. The importance of consideration of tautomeric states and ligand strain for glucose analogues in the confined space of the catalytic site with the 280s loop in the closed position was highlighted.

## 1. Introduction

Glycogen phosphorylase (GP; EC 2.4.1.1) is the rate-determining enzyme in the glycogenolysis pathway and a validated target for the development of new anti-hyperglycemic agents [1]. Diabetes is a major socio-economic burden with over 0.5 billion people living with diabetes worldwide, and with this predicted to increase by 643 million by 2030 and 783 million in 2045, the problem urgently requires more effective interventions that those currently available [2]. GP is a validated target for type 2 diabetes (T2D) and has considerable potential in this regard. A number of studies demonstrated the anti-diabetic potential of GP inhibitors in vivo [3,4]. Additionally, GP modulators have potential for treatment of other serious conditions such as myocardial and cerebral ischemias [5,6,7], and cancer [5,8]. Indeed, there was much recent interest with respect to control of glycogenolysis through GP inhibition for different cancers, that includes glioblastoma (GBM) [9,10]. GBM is the most common type of malignant brain tumour in adults and with a median survival rate measured in months (15 months) and 5-year survival of ~5% after initial diagnosis [11], novel strategies are crucial in the drug discovery pipeline. 

GP has three isoforms: liver, muscle and brain with 842–847 residues. It exists as a dimer and is an allosteric enzyme with a number of different binding sites, offering multiple opportunities for design of diverse chemotypes as GP inhibitors [12]. Studies on the catalytic, inhibitor, allosteric, new allosteric, quercetin binding [13], benzimidazole [14] and the glycogen storage sites were all reported. The GP enzyme exists in two states, the phosphorylated GPa form which is predominantly active (R state) and the unphosphorylated GPb form, which is predominantly inactive (T state) [1]. The inactive state is stabilized by the closed position of the 280s loop in the catalytic site.

**Scheme 1:**
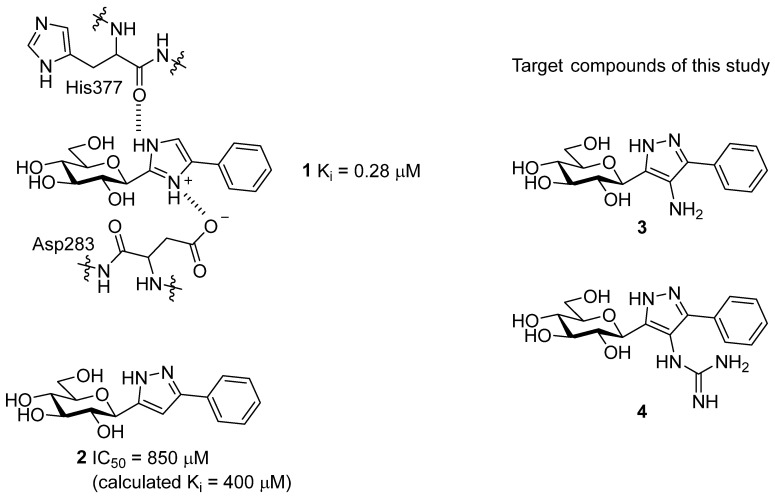
*C*-Glucopyranosyl imidazole **1**, with the important interactions in the catalytic site highlighted and pyrazole **2** type GP inhibitors, together with their efficacy against rabbit muscle GPb (*rm*GPb). The target compounds in this work are shown as **3** and **4**.

By far, the most explored binding site for GP inhibitor design is the catalytic site, where glucose analogues proved to be particularly effective [15,16]. The physiological inhibitor of GP is α-d-glucose with a *K_i_* of 1.7 mM; its anomer β-d-glucose has a *K_i_* of 7.4 mM [17]. Design of glucose analogues with carefully chosen β-substituents at the anomeric carbon were proven to be particularly effective, with the most successful inhibitor designs having a carefully chosen linker group between the glucose moiety and an aromatic group, the latter extending into the so-called β-cavity [15,18]. The chemotype of the linker is crucial to the inhibitory potential, where azole type heterocycles were proven to be particularly effective in recent work for inhibition of the isolated purified enzyme [19,20] and also in cellular experiments for reduction in glycogenolysis [19]. The most effective azole-linked inhibitors are those that can form a hydrogen bond with the His377 backbone C=O, whose presence is generally crucial to good activity. The imidazole linker revealed potent nanomolar inhibitors (e.g., **1** in Scheme 1 with a *K_i_* of 0.28 µM [21]) which, in addition, also proved to result in the first reported dual GP-SGLT inhibitors [22]. With the *pK_a_* for the protonated imidazole calculated as ~ 5.5–6.2, it was proposed that while the predominantly unprotonated neutral state favours cell permeation, on binding to GP, there is a shift in the equilibrium with the protonated state favoured [19]. The predicted binding of the phenyl analogue (**1**) demonstrated that the protonated heterocycle state forms the aforementioned important hydrogen bond with His377 C=O, but is also able to exploit favourable ion–ion interactions with the Asp283 from the 280s loop (Scheme 1), stabilizing the inactive state (closed position of the loop) [19]. 

Considering the binding features of **1**, it was speculated whether modifications of hydrogen bonding potential of an azole substituent with the Asp283 sidechain and/or its 3D arrangement in forming ion–ion interactions with the carboxylate sidechain group might be favourable, while maintaining the critical hydrogen bond interaction with His377 backbone C=O. In that regard, **3** and **4**—the C-4 substituted derivatives of pyrazole **2** (Scheme 1)—were considered for synthesis in this study. The pyrazole linker of **3** has a potential for both His377 C=O (as shown earlier [21]) and Asp283 side-chain hydrogen bonding, while **4** has the same hydrogen bonding potential but an additional possibility for ion–ion interactions with the Asp283 side-chain. In theory, the T state conformation of the enzyme would be favoured by stabilisation of the closed conformation of the 280s loop, blocking access of the substrate to the catalytic site. In this paper, we describe syntheses of compounds **3** and **4**, as well as their inhibitory potencies against rabbit muscle GPb (*rm*GPb). Additionally, extensive computations on the bound and unbound states of the ligands (Monte Carlo conformational searches, DFT, docking and post-docking molecular mechanics—generalized Born surface area (MM-GBSA)) are presented, to rationalize the observed binding assay results.

## 2. Results and Discussion

### 2.1. Synthesis

For the preparation of the target compounds, we chose a straightforward method based on the reaction of a sugar derived 1,3-dielectrophile and hydrazine. The C-N bond of the amino substituent of the heterocycle was formed at the level of dielectrophile, since we failed to produce the necessary intermediate from the known *O*-perbenzoylated 3-(β-d-glucopyranosyl)-5-phenyl-1*H*-pyrazole [21] by C-4 nitration/nitrosation of the pyrazole.

**Scheme 2:**
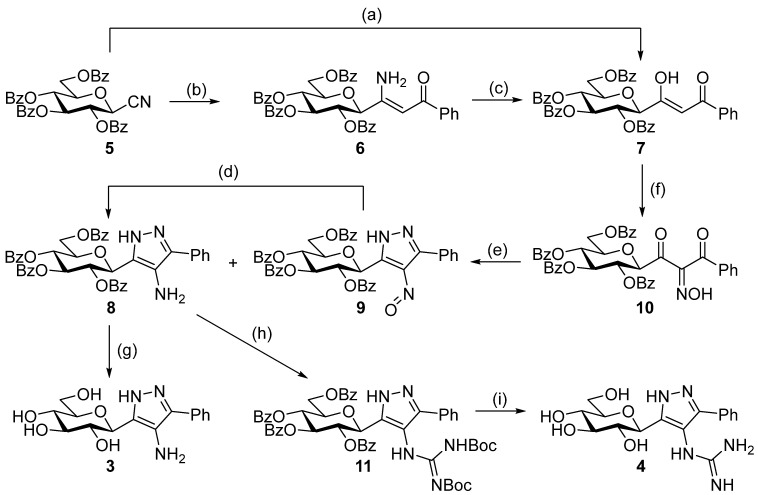
Synthesis of the target *C*-glucopyranosyl pyrazoles. Reagents and conditions: (a) Zn powder, Me_3_SiCl, phenacyl bromide, anh. THF, Ar, reflux, 2. 10% *w*/*w* aqueous HCl, 85 °C, 33%; (b) 1. Zn powder, Me_3_SiCl, phenacyl bromide, anh. THF, Ar, reflux, 2. 10% *w*/*w* aqueous HCl, 0 °C, 37%; (c) 10% *w*/*w* aqueous HCl, 85 °C, 27%; (d) H_2_, Pd(C), EtOAc, r.t., 74%; (e) N_2_H_4_·H_2_O, EtOH, 0 °C—r.t. **8**: 47%, **9**: 18%; (f) NaNO_2_, AcOH, H_2_O, 10 °C—r.t., 82%; (g) NaOMe, anh. MeOH, CHCl_3_, r.t., 58%; (h) *N*,*N′*-di-Boc-1*H*-pyrazole-1-carboxamidine, pyridine, r.t., 39%; (i) 1. CF_3_COOH, anisole, anh. DCM, r.t., 2. NaOMe, MeOH, r.t., 83%.

The dielectrophilic precursor was prepared from glucosyl cyanide **5** [23] with phenacyl bromide under Blaise conditions [24] (Scheme 2). Acidic workup at low temperature gave enaminone **6**, while hydrolysis of the Blaise reaction mixture at 85 °C gave enol **7**, which, upon treatment with NaNO_2_ under acidic conditions [25], furnished oxyme **10**. Ring closure of **10** with an excess of hydrazine monohydrate resulted in 4-aminopyrazole **8** as the main product together with the non-reduced 4-nitrosopyrazole **9**. The latter compound was identified based on its green colour; furthermore, the C-4 chemical shift difference of the nitrosated pyrazole **9** (157.1 ppm) compared to its non-nitrosated counterpart (101.4 ppm) [21] was 55.7 ppm, which is in good agreement with literature data [26]. Catalytic hydrogenation of the nitroso compound **9** led to the amino derivative **8**, from which we obtained one of the target compounds (**3**) via *O*-deprotection under Zemplén conditions. Treatment of **8** with *N*,*N′*-di-Boc-1*H*-pyrazole-1-carboxamidine [27] resulted in the protected guanidine derivative **11**, from which the other target molecule **4** was obtained after *N*-Boc and *O*-benzoyl cleavage.

### 2.2. Glycogen Phosphorylase Binding Assays

The inhibitory potency of **3** and **4** was assessed using binding assay experiments against *rm*GPb. The determined *IC*_50_ values are shown in Table 1, together with the previously determined *K_i_* value for benchmark compound **1**. Compound **3** (*IC*_50_ = 565 µM) was a moderate inhibitor of *rm*GPb and, while better than compound **2** (*IC*_50_ = 850 µM), it was much less potent than compound **1** (*K_i_* = 0.28 µM); compound **4** was revealed as a poor inhibitor (no inhibition at 625 µM). Towards understanding the observed potencies, extensive computations on the bound and unbound states of the ligands were performed.

### 2.3. In-Silico Studies

**Figure 1:**
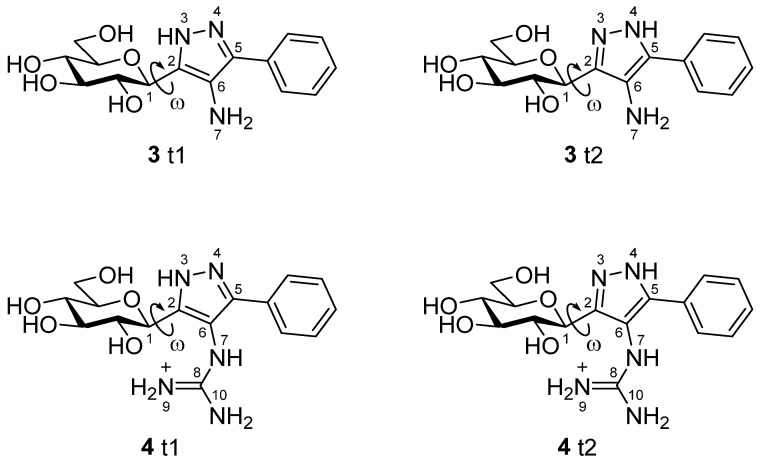
The predicted relevant ionization/tautomeric states of the unbound ligands based on LigPrep, Jaguar *pK_a_* and DFT calculations. Rotation around key dihedral angle ω (C^1^H-C^1^-C^2^-N^3^) for the different conformations is highlighted. Atoms are numbered for discussion in the text.

The binding assay results necessitated structural studies to elucidate the nature of protein–ligand interactions leading to the observed low potencies. Computational studies have proven to be a useful tool to rationalize GP inhibitor efficiency [28,29], including previously reported glucose analogues containing heterocyclic linkers [19,30,31]. As the initial design hoped to exploit interactions with the His377 backbone C=O and strong interactions with the Asp283 sidechain carboxylate stabilizing the closed position of the 280s loop, it was important to first establish the most stable states of the free unbound ligands, prior to the protein–ligand binding calculations. For this purpose, ionization and tautomeric state stabilities of the ligands [32] were explored using Monte Carlo conformational searches supplemented by DFT post-processing minimizations (M06-2X/6-31+G*) to determine the stable unbound conformations. In a very recent benchmarking study of drug-like scaffolds, the M06-2X method outperformed a range of semi-empirical and quantum mechanical methods in terms of accurate calculation of relative tautomeric energies [33]. Furthermore, the M06-2X/6-31+G* level of theory was previously successfully applied to study tautomeric states of glucose-azole type inhibitors [19]. The chemical structures of the relevant unbound states (ionization/tautomeric) of the ligands **3** and **4** are shown in Figure 1. In a study of tautomer preferences in PDB complexes, the most stable water state tautomer is predominantly the most favoured binding state tautomer, depending on the ∆G between the two tautomers [34]. Solution phase energies were calculated for the optimized DFT conformations using M06-2X/6-31+G* with solvation effects included with the SM8 water solvation model.

With respect to the protein–ligand bound states, the end point method MM-GBSA is recognised as an effective post-docking strategy for the calculation of relative binding free energies (∆*G_bind_*) [35]. Glide-SP docking poses of **3** and **4** were post-processed using MM-GBSA using two equations.

(1) $${∆G^{\prime }}_{bind}(NS)={∆E}_{MM}+{∆G}_{solv}$$

In Equation (1), $E_{MM}$ represents the total molecular mechanics energy (internal, electrostatic and van der Waals); $G_{solv}$, the solvation free energy calculated using the variable-dielectric generalized Born solvation model. As MM-GBSA is an endpoint method, it considers the differences Δ between the bound and unbound states of the complex, calculated with the OPLS3e forcefield, yielding a ${∆G^{\prime }}_{bind}(NS)$ in which strain/reorganisation effects on binding are neglected (NS = no strain). To further include both the ligand strain energy (protein was treated as rigid throughout), as well as an estimate for the loss of ligand vibrational, rotational and translational (VRT) entropy on binding, a corrected ${∆G}_{bind}$ was calculated by Equation (2) as follows:

(2) $${∆G}_{bind}={∆G^{\prime }}_{bind}(NS)+{Strain\;Energy}_{ligand}-{T∆S}_{MM}$$

Benchmark ligand **1** was also recalculated [19] and included for comparative purposes. 

**Figure 2:**
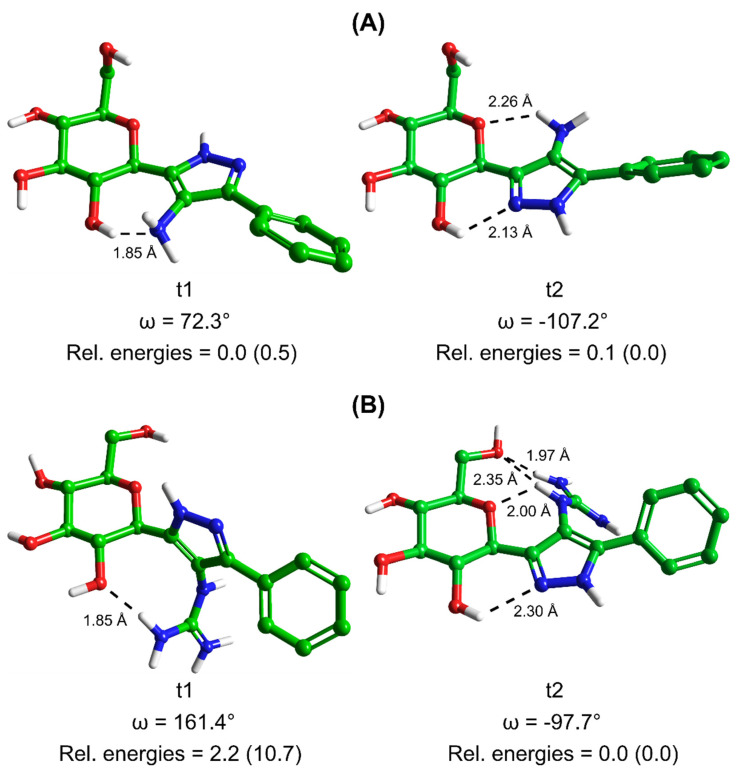
Predicted most stable conformations of tautomers 1 (**t1**) and 2 (**t2**) of compounds (**A**) **3** and (**B**) **4** (*c.f.* Figure 1) in solution phase from Monte Carlo simulations post-processed by DFT calculations. Intramolecular hydrogen bond distances are given and shown as dashed lines, together with the dihedral angle value ω (as defined in Figure 1). The solution phase relative energies of the two tautomers of each ligand are listed, with gas phase relative energies in parentheses for comparison.

**Figure 3:**
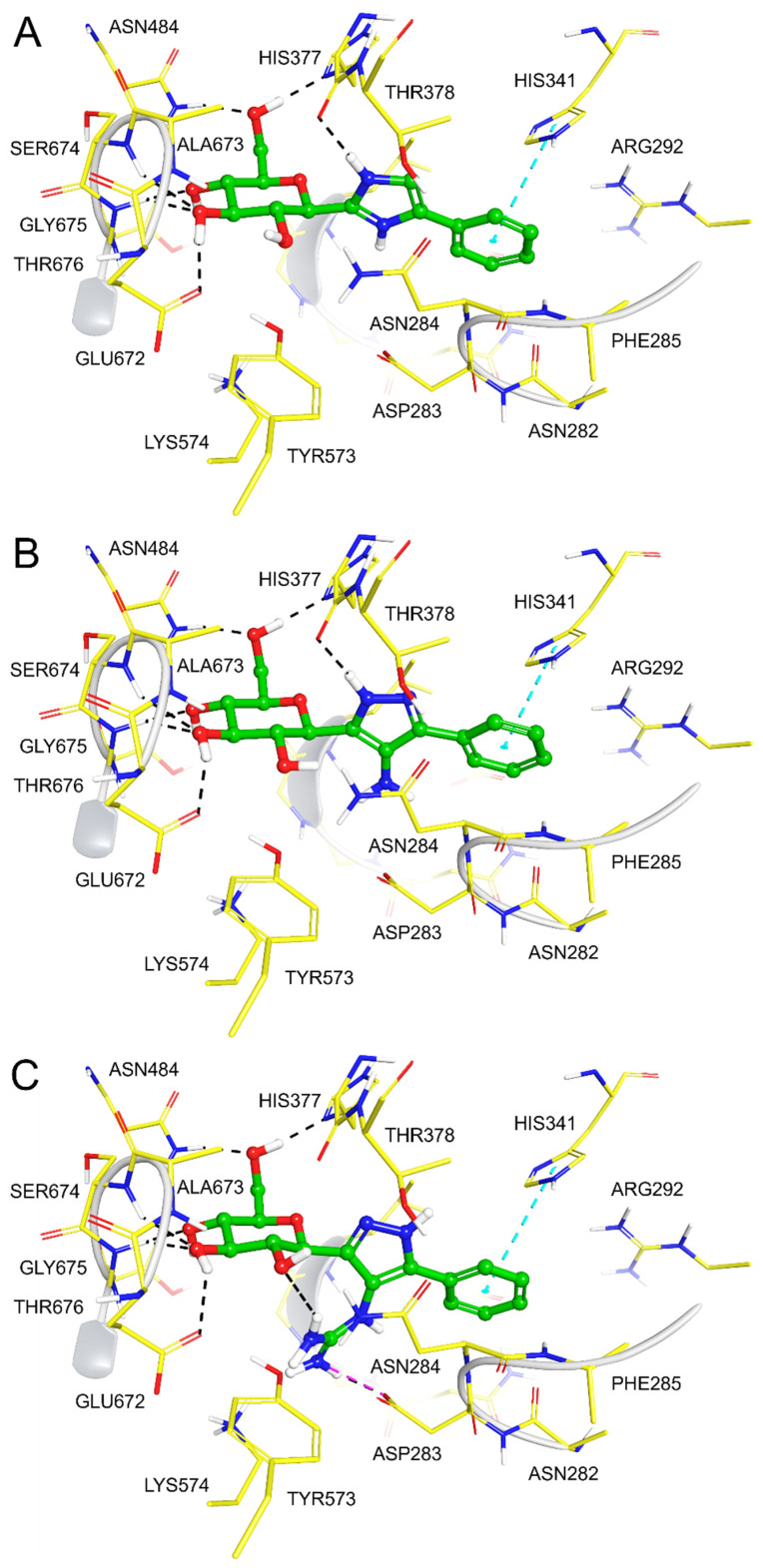
Binding modes of (**A**) **1**, (**B**) **3** and (**C**) **4** following post-docking MM-GBSA calculations.

**Analysis of 3**. Results of the DFT calculations for predicted conformations of compound **3** are shown in Table 2. The unbound state calculations for **3** revealed, as expected, that the -NH_2_ azole ring substituent would not be protonated using Jaguar *pK_a_* (predicted *pK_a_* for the protonated -NH3^+^ state was 4.69), as was also predicted by LigPrep [36]. There are two neutral tautomeric states **t1** and **t2** for compound **3** (Figure 1) and the calculated key dihedral angle ω (C^1^H-C^1^-C^2^-N^3^) for the lowest energy solution phase unbound conformation of each was ω = 72.3° and −107.2° (Table 2), respectively, from the DFT calculations. The same conformations were also the most stable in the gas phase. The most stable solution phase tautomer was predicted as **t1** (ω = 72.3°), but just by 0.1 kcal/mol. These most stable solution phase tautomeric conformations are shown in Figure 2A, where we can also see that intra-molecular hydrogen bonds stabilize the conformations and also, that the geometry around the -N(7)H_2_ substituent is not co-planar with its heterocycle. In line with expectations, the **t1** tautomer is the preferred binding tautomer from MM-GBSA calculations (Table 3), with a ∆*G_bind_* value of −40.9 kcal/mol (compared to −23.4 kcal/mol for **t2**). The ∆*G_bind_* value is, therefore, significantly less favourable than that of benchmark ligand **1** (−53.1 kcal/mol), which binds in the protonated state [19]. The predicted binding modes of both compounds **1** and **3** are shown in Figure 3, (A) and (B), respectively. Compound **1** (ω = −163.7°) exploits favourable hydrogen-bonding with His377 backbone C=O and ion–ion interactions with the Asp283 sidechain carboxylate; the bound state is consistent with its solved crystallographic complex (PDB code: 5JTT),with a ligand RMSD for heavy atoms of 0.152 Å. Compound **3** also adopts a conformation (ω = −173.0°) to have an N(3)H to His377 backbone C=O hydrogen bond. However, for **3,** there are no ion–ion interactions with Asp283 and the -N(7)H_2_ substituent cannot form direct hydrogen bonds with the carboxylate side-chain, although water-bridging interactions may be possible. Analysing the breakdown of contributions to ∆*G_bind_* (Table 3), it is these less favourable contacts as well as potential contributions from competing tautomeric states (the unbound state tautomeric energy differences are predicted as low, as mentioned just above) that is the likely source of the much lower potency of **3** compared to **1**; the ${∆G^{\prime }}_{bind}(NS)$ value is less favourable by 14.3 kcal/mol, while the strain energy and entropy contributions are more similar. 

**Analysis of 4**. Results of the DFT calculations for predicted conformations of compound **4** are shown in Table 4. In the case of **4**, the azole ring C6 substituent has a resonating +1 charge on N9/N10 atoms and hence, the potential to form strong ion – ion interactions with the Asp283 side-chain carboxylate, as was observed for **1**. As with **3**, compound **4** can exist in two tautomeric states **t1** and **t2** (Figure 1). DFT calculations on the different conformations of **t1** and **t2** revealed tautomer **t2** with a dihedral angle (ω = −97.7°) as the most favoured tautomeric state and conformation in solution phase (also in the gas phase). In comparison, the lowest energy **t1** solution phase conformation (ω = 161.4°) is 2.2 kcal/mol higher in energy (or 8.3 kcal/mol in the gas phase with ω = 131.7°). These most stable solution phase conformations are shown in Figure 2B. The most stable tautomer **t2** has a strong network of intra-molecular hydrogen bonds stabilizing the structure, but for a conformation and tautomeric state that is not consistent with forming desired hydrogen bonding with His377 C=O and ion – ion interactions with Asp283 sidechain carboxylate on binding to GP. As mentioned, in a study of ligand tautomeric preferences in PDB complexes, the most stable solution phase tautomer (in this case **t2**) is predominantly the binding state tautomer (depending on the ∆G between the two tautomers) [34]. In agreement with this study, and of particular significance, we observed for other β-d-glucopryranosyl-azole inhibitors, considering the binding and MM-GBSA ∆*G_bind_* values of the most stable (solution phase) free state tautomer gave better agreement with experiment [19]. The ∆*G_bind_* value of **4** (**t2**) is −28.2 kcal/mol (Table 3), much less favourable than that of **3** (**t1**; ∆*G_bind_* = −40.9 kcal/mol), which is consistent with the experimental binding assay results (Table 1). The binding of **t2** is shown in Figure 3C, where the ligand is seen to adopt the expected binding conformation (ω = −177.1°) that does allow for strong ion–ion and hydrogen bonding interactions with Asp283 sidechain carboxylate. However, there is no hydrogen bond with His377 C=O for this tautomer (${∆G^{’}}_{bind}(NS)$ = −67.9 kcal/mol). Additionally, the guanidino ligand substituent due to its steric bulk also extends somewhat towards the positively charged side-chain of Lys574, forming unfavourable ion–ion interactions with this group. Correspondingly, the strain energy effects on ∆*G_bind_* are considerably more (~+18 kcal/mol) compared to **3** (~+8 kcal/mol) or **1** (~+10 kcal/mol). The strain from Prime MM-GBSA calculations is estimated from a (local) minimization of bound ligand conformation. Likewise, the ligand entropy cost ($-{T∆S}_{MM})$ on binding for **4** is ~4 kcal/mol more than both **1** and **3**, all of which is consistent with its poor observed binding potential.

## 3. Conclusions

The search for potent GP inhibitors acting at the catalytic site has focussed on glucose analogues, with *C*-β-d-glucopyranosyl azole type inhibitors proving to be among the most successful. Two new analogues of this type, compounds **3** and **4**, were synthesized that, in theory, had the potential to exploit key interactions with key binding site residues His377 and Asp283, but only **3** demonstrated a moderate potency. Extensive computations were performed on the free/unbound (Monte Carlo, DFT) and bound state protein–ligand complexes (docking, MM-GBSA) and revealed tautomeric state preferences and ligand strain/reorganization energies as key reasons. We observed that taking the ∆*G_bind_* value for the most stable (unbound) state tautomer produced results more in line with the experiment, consistent with a previous work on related analogues [19]; however, this is likely to be system dependent and sensitive to relative tautomeric state stabilities (comparing bound and unbound) [34]. Although some experimental techniques such as neutron scattering can sometimes be employed to determine ligand-bound state tautomeric states, routine X-ray crystallography structures of protein–ligand complexes will not show the H-atom positions [37,38,39]. The importance of careful consideration of ligand tautomeric/ionization state preferences in structure-based inhibitor design using computation was, therefore, highlighted, as well as consideration of tautomeric state conformational preferences (bound versus unbound) that limits the reorganization/strain energy on binding. This information can be exploited in further studies of this type targeting GP, but also other drug targets where ionization/tautomerism of ligand designs plays an important role. 

## 4. Experimental Section

### 4.1. Synthetic Methods

Thin-layer chromatography was carried out on aluminium sheets coated with silica gel 60 F_254_. TLC plates were inspected under UV light and developed by spraying with a staining reagent (5% of cc. H_2_SO_4_ and 1% of *p*-anisaldehyde in EtOH) followed by heating. Column chromatography was performed on silica gel 60 (63–200 µm). Optical rotations were measured using a Perkin Elmer 241 polarimeter. ^1^H and ^13^C NMR spectra (supplementary materials) were recorded using Bruker DRX 360 or Bruker DRX 400 spectrometers with TMS (^1^H spectra in CDCl_3_) or the residual solvent peak (^1^H spectra in CD_3_OD, ^13^C spectra in CDCl_3_ and CD_3_OD) as the internal standard. Mass spectra were recorded using a Bruker maXis II UHR ESI-TOF MS spectrometer. Anhydrous THF was distilled from sodium benzophenone ketyl and then, stored over sodium wires. Anhydrous MeOH was prepared by distillation over Mg turnings and iodine. Anhydrous CHCl_3_ was dried by distillation from P_4_O_10_, and was then stored over 4Å molecular sieves. 

Pyrazole tautomerization results in signal broadening; therefore, the ^13^C peaks of the heterocycle (and, in one case, the anomeric carbon of the sugar) cannot be identified in the carbon spectrum. In these cases, HRMS confirms the presence of the pyrazole moiety in the molecules.

**(*Z*)-3-amino-3-(2,3,4,6-tetra-*O*-benzoyl-β-** **d** **-glucopyranosyl)-1-phenylprop-2-en-1-one (6)**


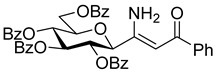


Prior to use, zinc powder was activated as described in the literature [40]. On a sintered glass funnel, zinc powder (100 mesh) was washed sequentially with 10% *w*/*w* aqueous HCl, distilled water, ethanol and diethyl ether and dried in a desiccator over P_2_O_5_.

In a flame dried three-neck round bottom flask, activated Zn powder (2 equiv., 1.65 mmol, 108 mg) and Me_3_SiCl (0.03 equiv., 0.03 mmol, 3 µL) were refluxed in anhydrous THF (4 mL) under argon atmosphere for 25 min. To this boiling suspension, the solution of cyanide (**5**, 1 equiv., 0.83 mmol, 500 mg) and phenacyl bromide (1.5 equiv., 1.24 mmol, 247 mg) in anhydrous THF (4 mL) was added dropwise in 45 min and the reflux was maintained for another 45 min. After cooling down to room temperature, the solution and the insoluble materials were separated by decantation and the residual solid was washed with THF (3 mL). The combined THF solutions were cooled down to 0 °C, 10% *w*/*w* aqueous HCl (4 mL) was added and the solution was stirred for 20 min at this temperature. Water (20 mL) was added and the mixture was extracted with DCM (3 × 20 mL). The combined organic layers were washed with saturated NaHCO_3_, dried over MgSO_4_, filtered and the solvent was removed. The resulting crude product was purified by column chromatography (eluent: hexane/EtOAc 2:1) to yield 223 mg (37%) colourless syrup.

R_f_ = 0.22 (hexane/EtOAc 2:1); [α]_D_ = −4 (c = 0.16, CHCl_3_)

^1^H NMR (360 MHz, CDCl_3_) δ (ppm): 9.93 (1H, brs, NH, exchangable) 8.07–7.83 (8H, m, Ar), 7.57–7.23 (15H, m, Ar), 7.09 (2H, t, *J* = 7.7 Hz, Ar), 6.04 (1H, pt, *J* = 9.6 Hz, H-2 or H-3 or H-4), 6.02 (1H, brs, NH, exchangable, overlaps with the previous signal), 5.87 (1H, pt, *J* = 9.8, 9.7 Hz, H-2 or H-3 or H-4), 5.71–5.66 (2H, m, H-2 or H-3 or H-4, C(NH_2_)=CHCOPh), 4.72 (1H, dd, *J* = 12.4, 2.6 Hz, H-6a), 4.56 (1H, dd, *J* = 12.4, 5.1 Hz, H-6b), 4.40 (1H, d, *J* = 9.6 Hz, H-1), 4.29 (1H, ddd, *J* = 9.6, 4.8, 2.6 Hz, H-5). ^13^C NMR (90 MHz, CDCl_3_) δ (ppm): 189.9 (C(NH_2_)=CHCOPh), 166.3, 165.9, 165.3, 164.7 (4 × OCOPh), 158.4 (C(NH_2_)=CHCOPh), 139.5 (Ar), 133.7–126.9 (Ar), 91.3 (C(NH_2_)=CHCOPh), 77.7, 76.4, 73.9, 71.1, 69.2 (C-1–C-5), 62.9 (C-6).

**(*Z*)-3-hydroxy-3-(2,3,4,6-tetra-*O*-benzoyl-β-** **d** **-glucopyranosyl)-1-phenylprop-2-en-1-one (7)**


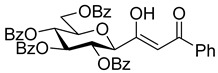


It was prepared from cyanide (**5**, 1 equiv., 0.83 mmol, 500 mg) in the same way as compound **6**, but the hydrolysis with aqueous HCl was carried out for 20 min at 85 °C. Yield: 195 mg (33%) colourless syrup.

Hydrolysis of enaminone **6** (657 mg, 0.91 mmol) in THF (10 mL) with 10% *w*/*w* aqueous HCl (1 mL) as described above gave 180 mg (27%) of **7**.

R_f_ = 0.47 (hexane/EtOAc 1:1); [α]_D_ = −61 (c = 0.90, CHCl_3_)

^1^H NMR (400 MHz, CDCl_3_) δ (ppm): 15.6 (1H, s, OH) 8.11–7.80 (8H, m, Ar), 7.58–7.24 (17H, m, Ar), 6.60 (1H, s, C(OH)=CHCOPh), 6.01 (1H, pt, *J* = 9.4 Hz, H-2 or H-3 or H-4), 5.78 (1H, pt, *J* = 9.6 Hz, H-2 or H-3 or H-4), 5.75 (1H, pt, *J* = 9.6 Hz, H-2 or H-3 or H-4), 4.80 (1H, dd, *J* = 12.3, 2.9 Hz, H-6a), 4.54 (1H, dd, *J* = 12.3, 4.9 Hz, H-6b), 4.40 (1H, d, *J* = 9.7 Hz, H-1), 4.25 (1H, ddd, *J* = 9.8, 4.8, 2.9 Hz, H-5). ^13^C NMR (100 MHz, CDCl_3_) δ (ppm): 190.9 (C(OH)=CHCOPh), 183.6 (C(OH)=CHCOPh), 166.3, 165.9, 165.3, 165.2 (4 × OCOPh), 134.1 (Ar), 133.7–127.4 (Ar), 93.9 (C(OH)=CHCOPh), 79.0, 76.4, 73.9, 70.7, 69.4 (C-1–C-5), 62.9 (C-6).

**2-Hydroxyimino-3-(2,3,4,6-tetra-*O*-benzoyl-β-** **d** **-glucopyranosyl)-1-phenylpropan-1,3-dione (10)**


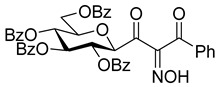


To the stirred solution of compound **7** (1 equiv., 0.28 mmol, 200 mg,) in AcOH (6 mL), aqueous solution (1 mL) of NaNO_2_ (6 equiv., 1.65 mmol, 114 mg) was added at 10 °C. The mixture was slowly allowed to warm up to room temperature in 1 h. After 15 min of stirring at room temperature, water (20 mL) was added and the solution was extracted with DCM (3 × 20 mL). The combined organic layers were dried over MgSO_4_; after filtration, the solvent was removed by evaporation and the residue was purified by column chromatography (eluent: hexane/EtOAc 2:1) to yield 171 mg (82%) yellowish syrup.

R_f_ = 0.32 (hexane/EtOAc 1:1); [α]_D_ = +8 (c = 1.03, CHCl_3_)

^1^H NMR (360 MHz, CDCl_3_) δ (ppm): 9.67 (1H, brs, NOH), 8.03–7.71 (8H, m Ar), 7.54–7.26 (17H, m, Ar), 6.02 (1H, pt, *J* = 9.4 Hz, H-2 or H-3 or H-4), 5.95 (1H, pt, *J* = 9.6 Hz, H-2 or H-3 or H-4), 5.72 (1H, pt, *J* = 9.6 Hz, H-2 or H-3 or H-4), 5.20 (1H, d, *J* = 9.6 Hz, H-1), 4.55 (1H, dd, *J* = 12.6, 2.6 Hz, H-6a), 4.43 (1H, dd, *J* = 12.0, 4.8 Hz, H-6b), 4.24–4.21 (1H, m, H-5). ^13^C NMR (90 MHz, CDCl_3_) δ (ppm): 190.4, 188.6 (COC(NOH)COPh), 166.4, 166.3, 165.3, 165.0 (4 × OCOPh), 155.2 (COC(NOH)COPh), 134.8, 134.4 (Ar), 133.7–128.5 (Ar), 77.3, 77.1, 74.5, 69.7, 69.5 (C-1–C-5), 63.1 (C-6).

**4-Amino-5-phenyl-3-(2,3,4,6-tetra-*O*-benzoyl-β-** **d** **-glucopyranosyl)-1*H*-pyrazole (8)**


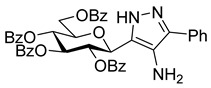


**4-Nitroso-5-phenyl-3-(2,3,4,6-tetra-*O*-benzoyl-β-** **d** **-glucopyranosyl)-1*H*-pyrazole (9)**


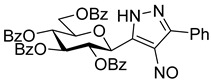


To the stirred solution of oxyme (**10**, 1 equiv., 0.07 mmol, 50 mg) in EtOH (1 mL), hydrazine monohydrate (10 equiv., 0.66 mmol, 32 µL) was added at 0 °C and the mixture was allowed to warm up to room temperature. After 1.5 h, DCM (5 mL) was added and the solution was washed with 1% *w*/*w* aqueous HCl (3 mL). The separated organic layer was dried over MgSO_4_, filtered, the solvent was removed by evaporation and the components of the mixture were separated by column chromatography (eluent: hexane/EtOAc 3:2) to yield **8** (23 mg, 47%, brownish solid) and **9** (9 mg, 18%, green solid).

Reduction in the nitroso derivative (**9**) was accomplished under general hydrogenation conditions in EtOAc (3 mL) over Pd(C) (15 mg of 10 wt.% Pd loading) under atmospheric pressure of hydrogen at room temperature overnight. 4-Aminopyrazole **8** was isolated by column chromatography (eluent: hexane/EtOAc 3:2) after filtration and evaporation of the reaction mixture. Yield: 40 mg (74%).

**8**: R_f_ = 0.27 (hexane/EtOAc 1:1); [α]_D_ = −65 (c = 1.65, CHCl_3_)

^1^H NMR (360 MHz, CDCl_3_) δ (ppm): 8.04–7.80 (8H, m Ar), 7.55–7.22 (17H, m, Ar), 6.13–6.03 (2H, m, H-2 and/or H-3 and/or H-4), 5.87 (1H, pt, *J* = 9.5 Hz, H-2 or H-3 or H-4), 5.11 (1H, d, *J* = 9.3 Hz, H-1), 4.68 (1H, dd, *J* = 12.2, 2.6 Hz, H-6a), 4.52 (1H, dd, *J* = 12.2, 4.5 Hz, H-6b), 4.36–4.33 (1H, m, H-5), 3.37 (2H, brs, NH_2_). ^13^C NMR (90 MHz, CDCl_3_) δ (ppm): 166.3, 166.0, 165.3, 165.1 (4 × OCOPh), 153.4 (pyrazole C-4), 133.5–125.0 (Ar), 76.6, 75.5, 74.6, 70.5, 69.6 (C-2–C-5), 63.2 (C-6).

**9**: R_f_ = 0.42 (hexane/EtOAc 1:1); [α]_D_ = +15 (c = 0.48, CHCl_3_)

^1^H NMR (360 MHz, CDCl_3_) δ (ppm): 8.03–7.69 (10H, m Ar), 7.53–7.13 (15H, m, Ar), 6.25 (1H, pt, *J* = 9.6 Hz, H-2 or H-3 or H-4), 6.11 (1H, pt, *J* = 9.4 Hz, H-2 or H-3 or H-4), 6.02 (1H, pt, *J* = 9.3 Hz, H-2 or H-3 or H-4), 5.25 (1H, d, *J* = 10.0 Hz, H-1), 4.58–4.48 (3H, m, H-6a, H-6b, H-5). ^13^C NMR (90 MHz, CDCl_3_) δ (ppm): 166.4, 166.3, 165.7, 165.2 (4 × OCOPh), 157.1 (pyrazole C-4), 133.7–127.2 (Ar), 75.2, 73.6, 70.9, 69.8, 69.5 (C-2–C-5), 63.4 (C-6).

**4-Amino-3-(β-** **d** **-glucopyranosyl)-5-phenyl-1*H*-pyrazole (3)**


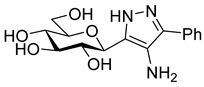


*O*-Perbenzoylated compound **8** (150 mg, 0.20 mmol) was dissolved in a mixture of anhydrous methanol (2 mL) and chloroform (2 mL), and pH was adjusted to 10 with NaOMe (1M in MeOH). The reaction mixture was stirred at room temperature overnight and then the solution was neutralized using Amberlyst 15 acidic ion exchange resin until pH 7. The resin was filtered off and the filtrate was concentrated. The product was purified by column chromatography (eluent: CHCl_3_/MeOH 8:2) to yield 38 mg (58%) colourless syrup.

R_f_ = 0.24 (CHCl_3_/MeOH 7:3); [α]_D_ = −1 (c = 1.35, MeOH)

^1^H NMR (400 MHz, CD_3_OD) δ (ppm): 7.62 (2H, m Ar), 7.44 (2H, t, *J* = 7.7 Hz, Ar), 7.33 (1H, m, Ar), 4.45 (1H, d, *J* = 9.6 Hz, H-1), 3.89 (1H, dd, *J* = 12.0, 2.2 Hz, H-6a), 3.76 (1H, dd, *J* = 12.0, 4.9 Hz, H-6b), 3.76 (1H, pt, *J* = 9.1 Hz, H-2 or H-3 or H-4), 3.55–3.48 (2H, m, H-2 and/or H-3 and/or H-4), 3.45–3.41 (1H, m, H-5). ^13^C NMR (100 MHz, CD_3_OD) δ (ppm): 129.9, 128.6, 127.7, 124.5 (Ar), 82.0, 79.6, 76.2, 74.9, 71.2 (C-1–C-5), 62.6 (C-6).

HRMS (positive mode, m/z): 344.1216 (calculated value for C_15_H_19_N_3_O_5_Na: 344.1217)

**4-(2,3-Bis(*tert*-butoxycarbonyl)guanidino)-5-phenyl-3-(2,3,4,6-tetra-*O*-benzoyl-β-** **d** **-glucopyranosyl)-1*H*-pyrazole (11)**


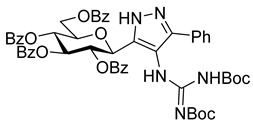


The solution of **8** (280 mg, 0.38 mmol) and *N*,*N′*-di-Boc-1*H*-pyrazole-1-carboxamidine (2 equiv., 0.76 mmol, 236 mg) in pyridine (3 mL) was stirred at room temperature for 10 days. After evaporation of the reaction mixture, the product was isolated by column chromatography (eluent: hexane/acetone 4:1 → 3:1) as a colourless syrup. Yield: 146 mg (39%). 

R_f_ = 0.56 (hexane/acetone 1:1); [α]_D_ = +19 (c = 0.70, CHCl_3_)

^1^H NMR (360 MHz, CDCl_3_) δ (ppm): 11.48 (1H, brs, NH), 9.86 (1H, brs, NH), 7.97–7.79 (8H, m Ar), 7.47–7.21 (17H, m, Ar), 6.01–5.94 (2H, m, H-2 and/or H-3 and/or H-4), 5.77 (1H, pt, *J* = 9.5 Hz, H-2 or H-3 or H-4), 5.38 (1H, d, *J* = 8.6 Hz, H-1), 4.65 (1H, dd, *J* = 12.2, 3.4 Hz, H-6a), 4.57 (1H, dd, *J* = 12.2, 4.4 Hz, H-6a), 4.26 (1H, m, H-5), 1.50 (9H, s, OC(CH_3_)_3_), 1.35 (9H, s, OC(CH_3_)_3_).

^13^C NMR (90 MHz, CDCl_3_) δ (ppm): 166.3, 166.1, 165.3, 165.2 (4 × OCOPh), 163.2 (NHC(=NBoc)NHBoc), 154.8, 153.2 (2 × NC(=O)O*t*Bu), 140.4, 140.2 (pyrazole C-2, C-5), 133.4–127.3 (Ar), 114.1 (pyrazole C-4), 83.9, 79.6 (2 × OC(CH_3_)_3_), 76.5, 74.9, 74.3, 71.2, 70.2 (C-1–C-5), 63.8 (C-6), 28.2, 28.1 (2 × OC(CH_3_)_3_).

HRMS (positive mode, m/z): 1002.3535 (calculated value for C_54_H_53_N_5_O_13_Na: 1002.3532)

**3-(β-** **d** **-Glucopyranosyl)-4-guanidino-5-phenyl-1*H*-pyrazole (4)**


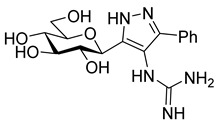


Protected pyrazole (**11**, 146 mg, 0.15 mmol) was dissolved in anhydrous dichloromethane (1.5 mL), anisole (324 µL, 20 equiv.) and trifluoroacetic acid (1.5 mL) were added and the mixture was stirred at room temperature. After 2 h, the volatiles were removed, the residue was dissolved in anhydrous methanol (3 mL) and NaOMe was added (1M in methanol) until pH 9. The cleavage of the benzoyl groups took place in 3 h at room temperature. The solution was neutralized using Amberlyst 15 resin and the resin was filtered and the filtrate was concentrated. The residue was purified by column chromatography on aluminium oxide (neutral, Brockmann I, 40–300 µm, 60A, eluent: MeCN/water/isopropyl alcohol/toluene 3:1.5:3.5:2) to yield 45 mg (83%) colourless syrup. 

R_f_ = 0.24 (DCM/MeOH/NH_3_ (25% *w*/*w* in water) 3:4:3); [α]_D_ = +5 (c = 2.9, MeOH)

^1^H NMR (400 MHz, CD_3_OD) δ (ppm): 7.67–7.65 (2H, m Ar), 7.50–7.40 (3H, m, Ar), 4.37 (1H, d, *J* = 9.6 Hz, H-1), 3.89 (1H, d, *J* = 11.9 Hz, H-6a), 3.76–3.68 (2H, m, H-6b, H-2 or H-3 or H-4), 3.53 (1H, pt, *J* = 8.3 Hz, H-2 or H-3 or H-4), 3.50–3.42 (2H, m, H-2 and/or H-3 and/or H-4, H-5). ^13^C NMR (100 MHz, CD_3_OD) δ (ppm): 159.3 (NHC(=NH)NH_2_), 130.1, 127.9 (Ar), 112.6 (pyrazole C-4), 82.0, 79.4, 74.6, 71.4 (C-2–C-5), 62.7 (C-6).

HRMS (positive mode, m/z): 386.1434 (calculated value for C_16_H_21_N_5_O_5_Na: 386.1435)

### 4.2. Computational Details

#### 4.2.1. Protein Preparation

The GPb protein was prepared for computations using the solved 1.85 Å resolution co-crystallized complex with compound **1** (PDB code: 5JTT, 1.85 Å resolution [41]) and Schrödinger’s Protein Preparation Wizard [36]. The waters within 5 Å of the native ligand were initially retained, bond orders assigned and hydrogens added, and the protonation states for basic and acidic residues assigned using PROPKA calculated p*K*_a_’s at a pH of 7 [42]. The subsequent optimization of hydroxyl groups, histidine protonation states and their potential side-chain C/N atom flips, as well as the side-chain O/N atom flips of Gln and Asn residues was based on optimizing protein hydrogen bonding patterns. The pyridoxal-phosphate (PLP) co-factor had the phosphate group assigned in a monoanionic state. Lastly, the system was gently minimised using the OPLS3e forcefield [43] but with the RMSD (heavy atoms) kept to within 0.3 Å of the crystallographic positions.

#### 4.2.2. Ligand Preparation

Ligands for the calculations were prepared using Maestro and LigPrep 5.6 [36] with the tautomeric and ionization states assigned based on the default pH of 7 +/− 2. Preferred protonation states of the heterocycles were also considered the using Jaguar *pK_a_* predictions [36]. The generated states were all used in the docking and post-docking calculations. To more accurately consider the relative stabilities of the free unbound ligand tautomeric states [33], DFT gas phase optimizations (Jaguar 11.0 [36]) were performed at the M06-2X/6-31+G* [44,45,46] level of theory, given its previous successful application to analogues of this type [19]. Input structures for these DFT calculations were based on Monte Carlo conformational searches using Macromodel 13.0 [36], with the predicted 10 lowest energy conformations for each ligand tautomeric state used. The conformational search employed 20,000 steps of the Monte Carlo Multiple Minima (MCMM) approach; each step was accompanied by a 100 steps minimization with the truncated Newton conjugate gradient (TNCG) algorithm; OPLS3e forcefield was employed, together with the Generalized-Born/Surface-Area (GB/SA) model for water solvation effects. For the DFT-optimized gas phase geometries, solution phase single point energy calculations were performed, with water solvation effects included with the SM8 continuum model [47].

#### 4.2.3. Docking 

Docking of the ligands was performed using the program Glide 8.9 [36]. Using the prepared GPb protein from PDB code:5JTT, the shape and properties of the catalytic site were mapped onto a grid with dimensions 24.3 × 24.3 × 24.3 Å that was centred on the cognate ligand (**1**). Docking positional constraints were placed on the glucopyranosyl hydroxyl hydrogens (radius 1.0 Å) to maintain the well-defined consistent position of the moiety from crystallographic studies. Otherwise, standard parameters were employed that included default atomic charges and van der Waals scaling (0.8) for nonpolar ligand atoms to include modest induced-fit effects. 

Calculations were performed in SP mode and included post-docking minimization with strain correction. Up to 5 output poses per ligand structure docked were saved. Redocking of the cognate ligand gave a top-ranked ligand pose with RMSD (heavy atoms) of just 0.152 Å compared to in the co-crystallized complex, for an initial validation of the applied protocol. 

#### 4.2.4. MM-GBSA Calculations

The docking poses for each ligand structure were used as input for post-docking Prime MM-GBSA 3.0 binding free energy calculations [36], which were calculated using Equations (1) and (2) described earlier, with Equation (2) accounting for the effects of ligand strain and entropy effects. The ${Strain\;Energy}_{ligand}$ was calculated by minimization of the bound ligand and ${T∆S}_{MM}$ was calculated using the Rigid Rotor Harmonic Oscillator approximation with MacroModel 13.0 and the OPLS_2005 forcefield [36]. All output Glide-SP docking poses for each input ligand structure were used for these calculations and the best ${∆G}_{bind}$ values taken as the predicted value for each compound.

#### 4.2.5. Determination of Inhibitory Constants (K_i_) for Glycogen Phosphorylase

Enzyme activity was assayed into the direction of glycogen synthesis as previously presented [22]. Kinetic data were collected using the muscle phosphorylase *b* (dephosphorylated, GP*b*) isoform. Kinetic data for the inhibition of GP*b* by the compounds were obtained in the presence of 10 μg/mL enzyme, varying concentrations of α-d-glucose-1-phosphate (4–40 mM), constant concentration (1%) of glycogen and AMP (1 mM). Enzymatic activities were presented in the form of a double-reciprocal plot (Lineweaver-Burk). The plots were analysed by a non-linear data analysis program. The inhibitor constants (*K_i_)* were determined by secondary plots, replotting the slopes from the Lineweaver-Burk plot against the inhibitor concentrations. The means of standard errors for all calculated kinetic parameters averaged to less than 10% [48,49].

## Figures and Tables

**Table 1:** Results from the binding assay experiments for the target compounds **3** and **4** against rabbit muscle glycogen phosphorylase b (*rm*GPb). Reference compounds **1** and **2** are shown for comparison.

Compound		IC_50_ [µM]
**1** ^a^	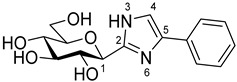	0.28 (*K_i_*) [21]
**2**	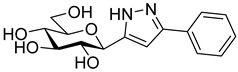	850 [21]
**3**	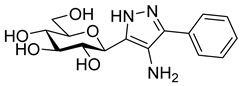	565
**4**	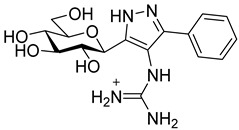	no inhibition at 625 µM

^a^ Atoms numbered for discussion in text.

**Table 2:** Relative energies (kcal/mol) of the predicted conformations of compound **3** tautomers **t1** and **t2** calculated using DFT at the M06-2X/6-31+G* level of theory (gas phase), with water solvation effects additionally accounted for with the SM8 model (solution phase). The most stable calculated state/conformation of the compound is highlighted in **bold** for each phase.

Conformation ^a^	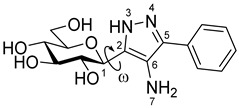 3 tautomer 1 (t1)			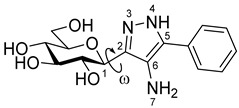 3 tautomer 2 (t2)		
	Dihedral Angle, ω (Degrees) ^b^	Relative Energies (kcal/mol)		Dihedral Angle, ω (Degrees) ^b^	Relative Energies (kcal/mol)	
		Gas Phase	Solution Phase ^c^		Gas Phase	Solution Phase ^c^
1	66.6	1.9	1.7	−104.8	0.3	0.7
2	65.8	1.1	0.6	−114.9	0.8	1.1
3	147.6	4.6	4.3	−89.9	1.5	1.2
4	118.0	2.0	2.5	−102.0	0.9	0.4
5	100.8	1.9	1.4	−100.7	0.7	0.6
6	82.7	2.2	1.4	**−107.2**	**0.0**	0.1
7	95.4	0.7	0.7	−88.7	2.0	1.8
8	**72.3**	0.5	**0.0**	−100.1	0.8	0.8
9	141.3	7.3	5.6	−135.9	8.4	5.1
10	84.3	2.2	1.5	−164.5	8.9	4.8

^a^ Conformations were predicted using Monte Carlo prior to optimizations using DFT, as described in the computational details. ^b^ Dihedral C^1^H-C^1^-C^2^-N^3^. ^c^ Single point energy calculations at the M06-2X/6-31+G* optimized geometries.

**Table 3:** Results of MM-GBSA post-docking calculations with the predicted binding tautomer/state highlighted in ***bold italics***.

Ligand and State	Dihedral Angle, ω C^1^H-C^1^-C^2^-N^3^ (Degrees) ^a^	Post-Docking MM-GBSA (kcal/mol) ^b^			
		${∆G^{\prime }}_{bind}(NS)$	${Strain\;Energy}_{ligand}$	$-{T∆S}_{MM}$	${∆G}_{bind}$
1 ^c^					
t1	−167.5	−64.8	10.1	18.8	−35.9
t2	−146.5	−51.7	10.7	18.9	−22.1
***+1 charged***	***−163.7***	***−81.4***	***10.2***	***18.1***	***−53.1***
3					
***t1***	***−173.0***	***−67.1***	***7.9***	***18.3***	***−40.9***
t2	−170.3	−52.8	11.4	18.0	−23.4
4					
t1	−166.2	−82.5	18.3	20.7	−43.5
***t2***	***−177.1***	***−67.9***	***17.7***	***22.0***	***−28.2***

***^a^*** Dihedral angle ω in the bound state. *c.f.* Table 1, Table 2 and Table 4 for dihedral atoms of compounds **1**, **3** and **4**, respectively. ^b^ *c.f.* Equations (1) and (2). ^c^ *c.f.* Table 1, **t1** = N^3^H; **t2** = N^6^H; +1 charged = N^3^H and N^6^H.

**Table 4:** Relative energies (kcal/mol) of the predicted conformations of compound **4** tautomers **t1** and **t2** calculated using DFT at the M06-2X/6-31+G* level of theory (gas phase), with water solvation effects additionally accounted for with the SM8 model (solution phase). The most stable calculated state of the compound is highlighted in **bold** for each phase.

Conformation ^a^	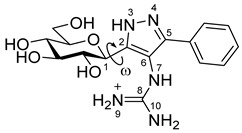 4 tautomer 1 (t1)			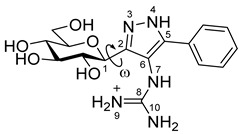 4 tautomer 2 (t2)		
	Dihedral Angle, ω (Degrees) ^b^	Relative Energies (kcal/mol)		Dihedral Angle, ω (Degrees) ^b^	Relative Energies (kcal/mol)	
		Gas Phase	Solution Phase ^c^		Gas Phase	Solution Phase ^c^
1	160.9	10.7	2.4	−141.7	3.9	0.9
2	9.8	17.0	5.0	−120.6	10.2	4.0
3	131.7	8.3	3.0	**−97.7**	**0.0**	**0.0**
4	132.6	11.7	5.7	−140.9	3.7	0.4
5	−154.2	9.7	4.0	−32.9	6.4	3.2
6	161.4	10.7	2.2	−178.2	13.2	2.1
7	−155.2	9.8	4.3	−144.1	5.5	2.7
8	158.8	10.7	3.4	175.8	10.0	1.5
9	−19.1	10.4	6.2	−143.4	5.1	2.1
10	159.6	13.6	3.5	−99.0	3.2	0.2

^a^ Conformations were predicted using Monte Carlo prior to optimizations using DFT, as described in the computational details. ^b^ Dihedral C^1^H-C^1^-C^2^-N^3^. ^c^ Single point energy calculations at the M06-2X/6-31+G* optimized geometries.

## Data Availability

All data appear in the article.

## Supplementary Materials

The following supporting information can be downloaded at: https://www.mdpi.com/article/10.3390/molecules28073005/s1. Copies of the ^1^H and ^13^C NMR spectra.

## Sample Availability

Samples of the compounds are not available.
