# Supplementary material for: Design and Synthesis of 3-(β-d-Glucopyranosyl)-4-amino/4-guanidino Pyrazole Derivatives and Analysis of Their Glycogen Phosphorylase Inhibitory Potential
Source: Molecules. 2023 Mar 28;28(7):3005. doi: 10.3390/molecules28073005 (PMC10095824; doi:10.3390/molecules28073005)

Supporting information

Design and Synthesis of 3-(β-D-Glucopyranosyl)-4-amino/4-guanidino Pyrazole Derivatives and Analysis of their Glycogen Phosphorylase Inhibitory Potential

Sándor Kun,[a] Rachel T. Mathomes,[b] Tibor Docsa,[c] László Somsák*[a] and Joseph M. Hayes*[b]

[a] Department of Organic Chemistry, University of Debrecen, POB 400, H-4002 Debrecen, Hungary

[b] School of Pharmacy & Biomedical Sciences, University of Central Lancashire, Preston PR1 2HE, United Kingdom

[c] Department of Medical Chemistry, University of Debrecen, Faculty of Medicine, University of Debrecen, 4032 Debrecen, Hungary

* Corresponding authors

tel: +441772894334 (JMH); email: jhayes@uclan.ac.uk

tel: +3652512900 ext. 22348 (LS), email: somsak.laszlo@science.unideb.hu

Copies of the ^{1}H and ^{13}C J-MOD NMR spectra

^{1}H (400 MHz) and ^{13}C J-MOD (100 MHz) NMR spectra of compound **3** in CD_3OD

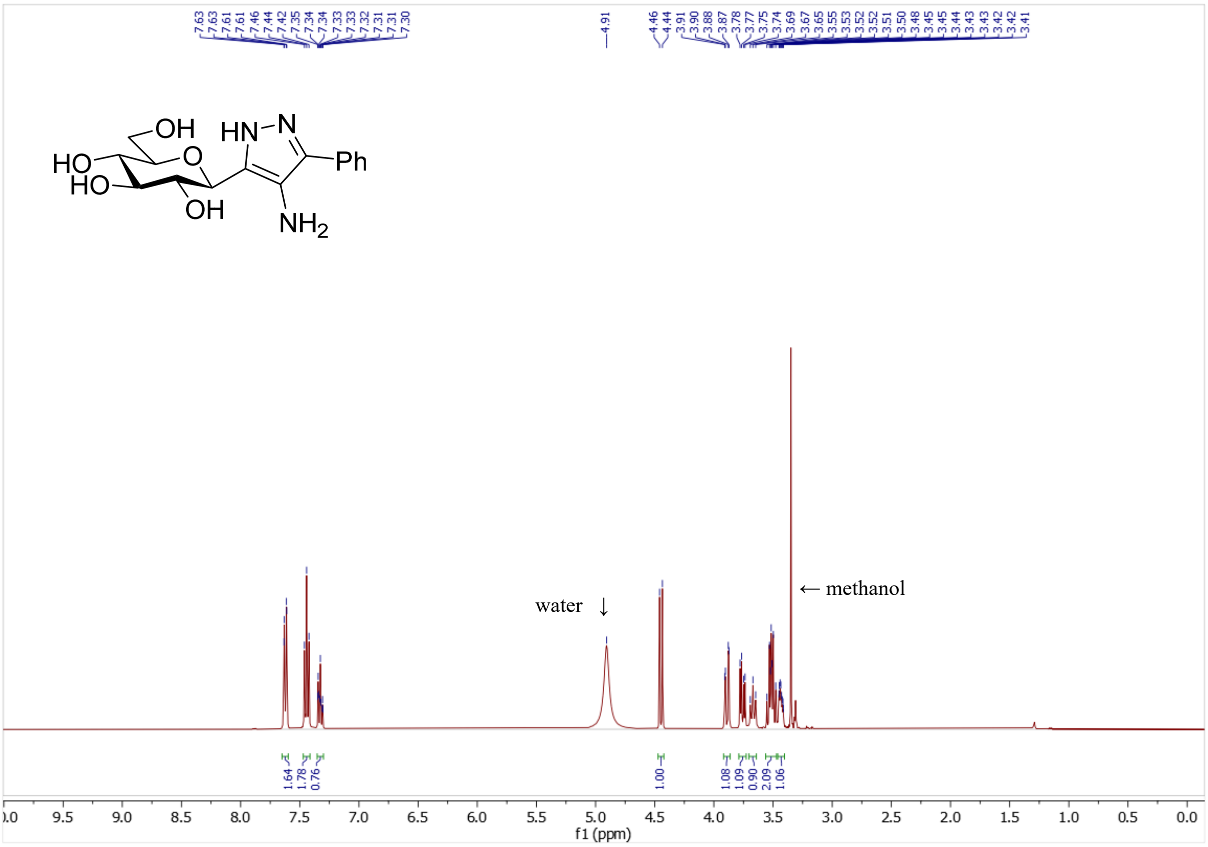

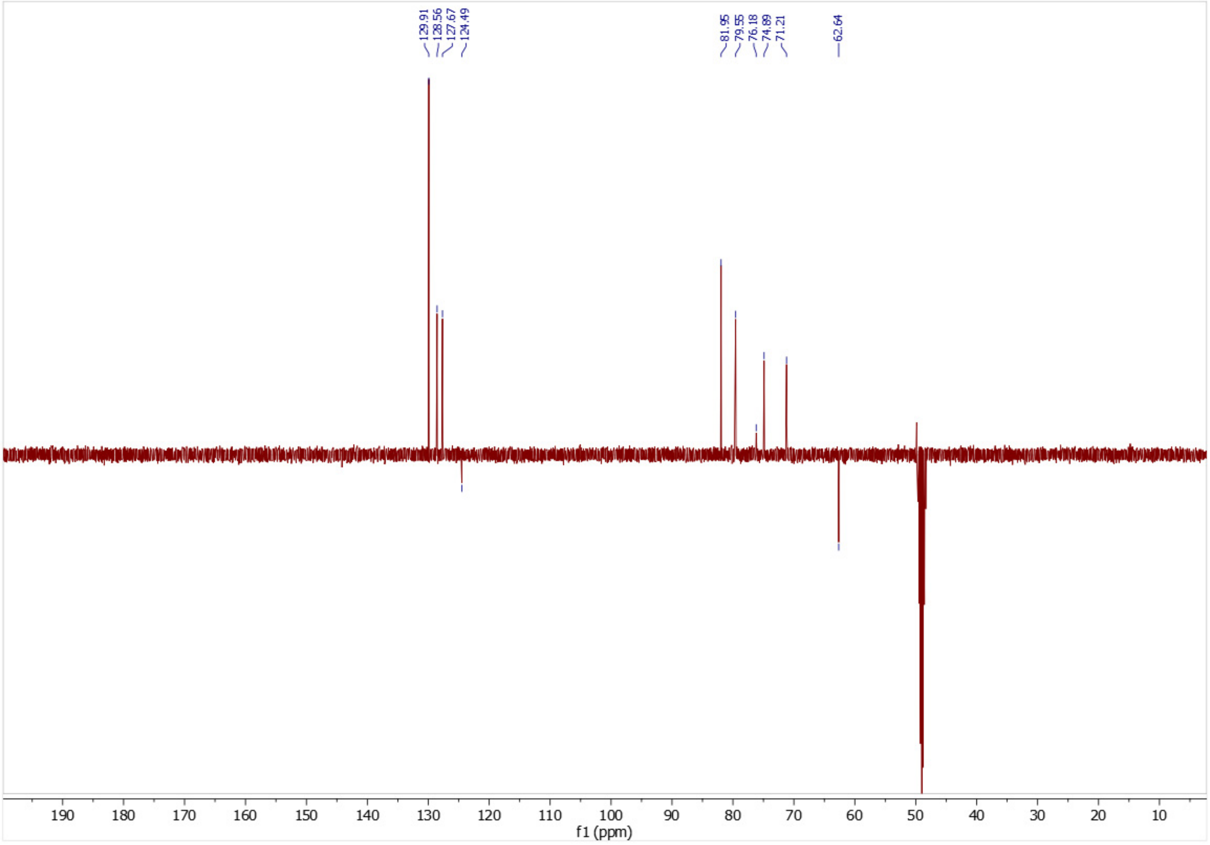

^{1}H (400 MHz) and ^{13}C J-MOD (100 MHz) NMR spectra of compound **4** in CD_3OD

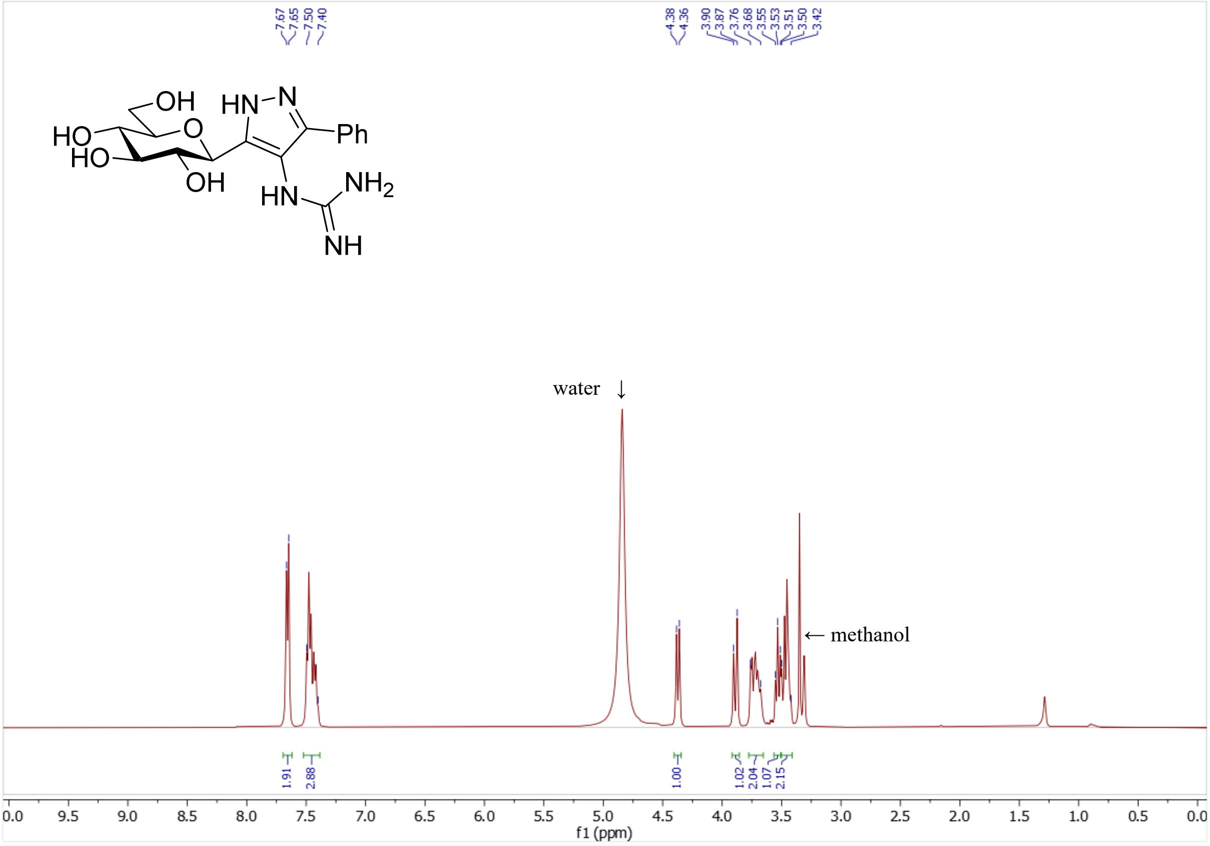

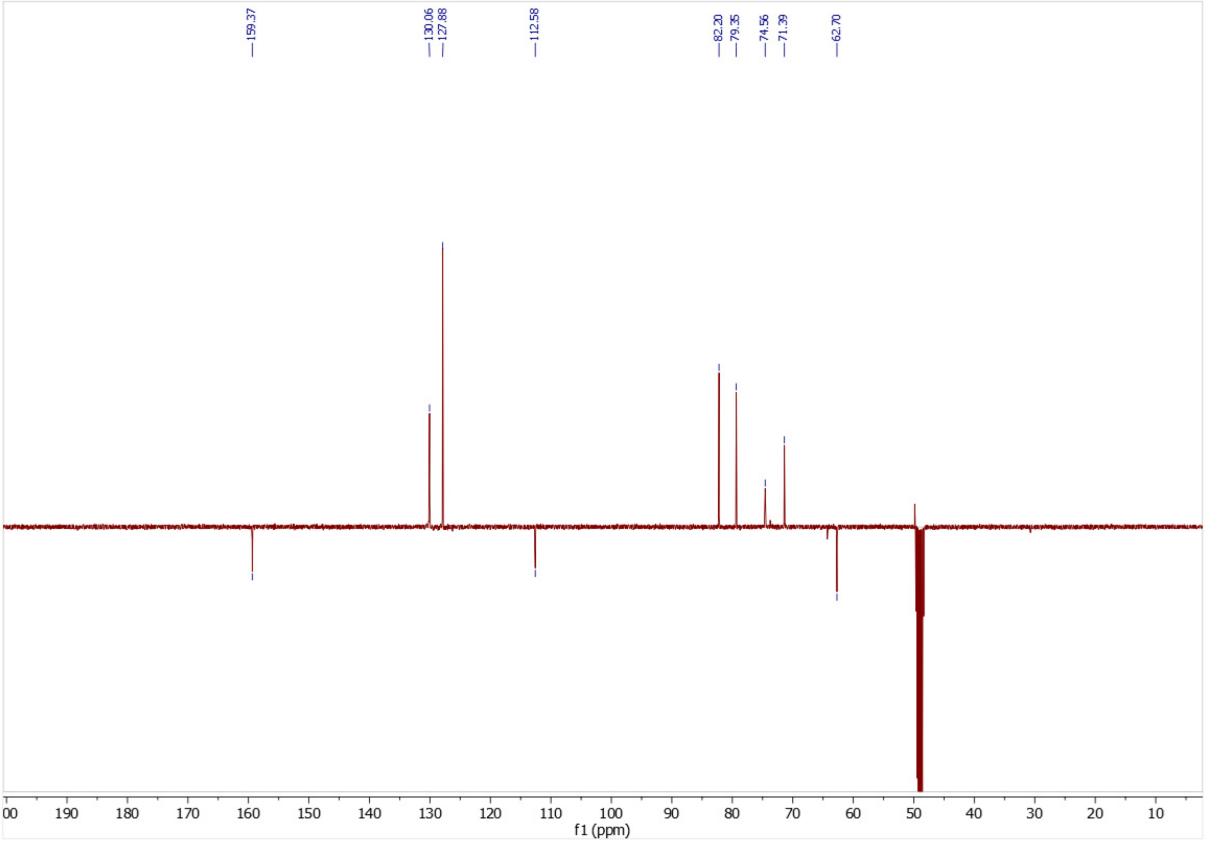

^{1}H (360 MHz) and ^{13}C J-MOD (90 MHz) NMR spectra of compound **6** in $CDCl_3$

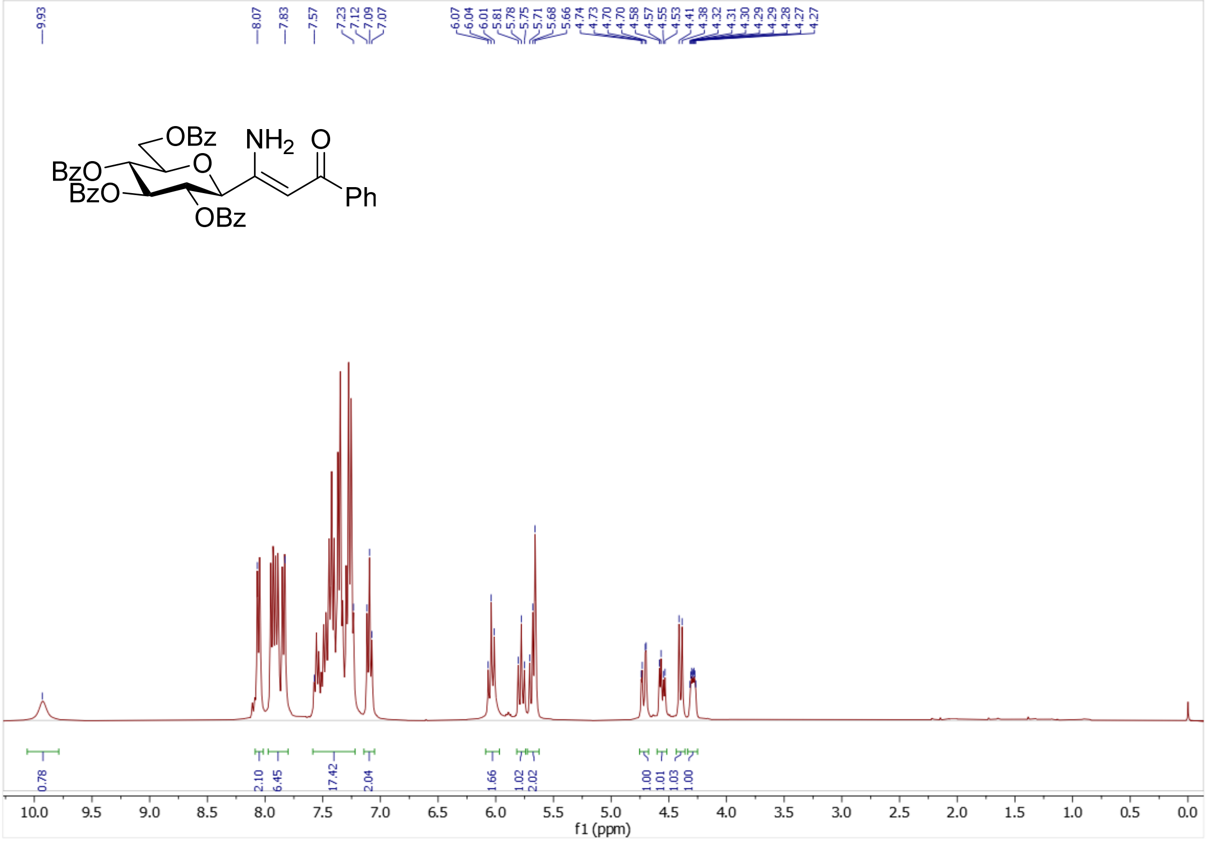

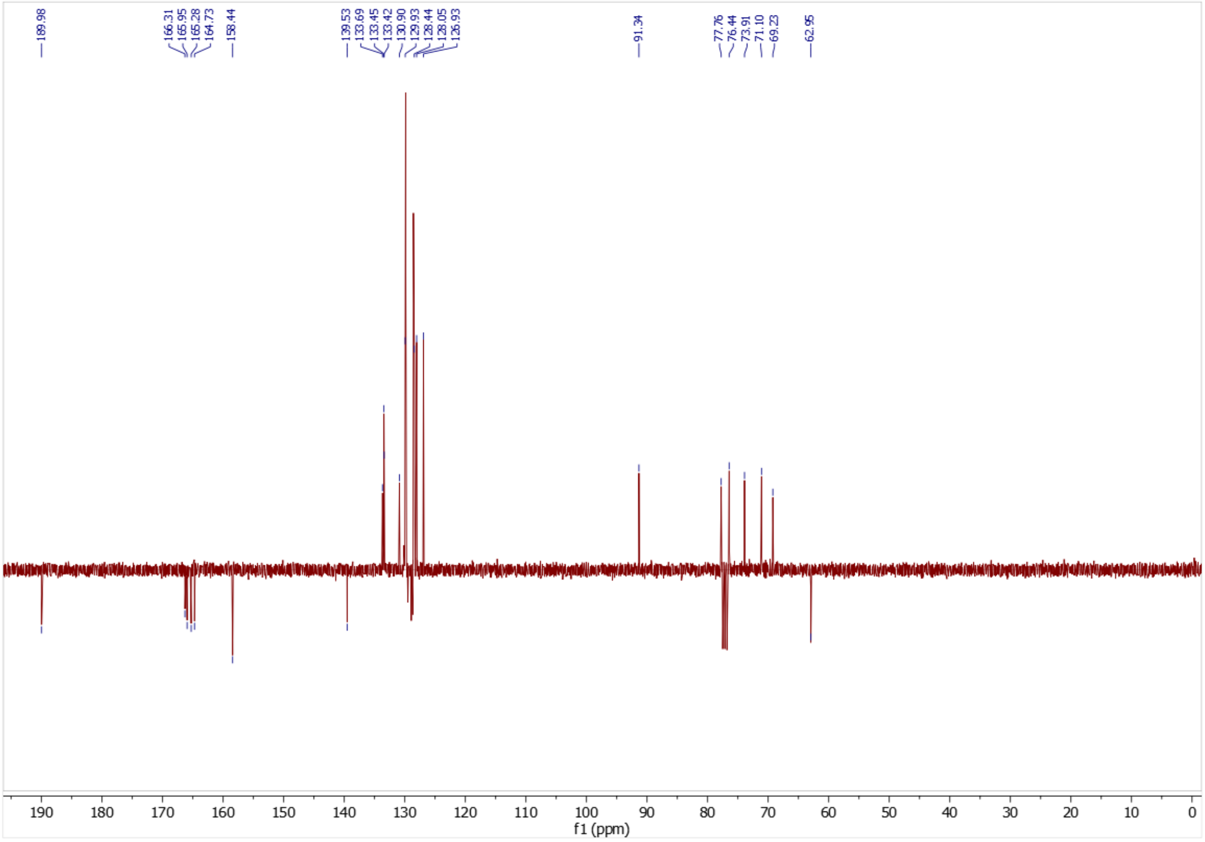

^{1}H (400 MHz) and ^{13}C J-MOD (100 MHz) NMR spectra of compound **7** in $CDCl_3$

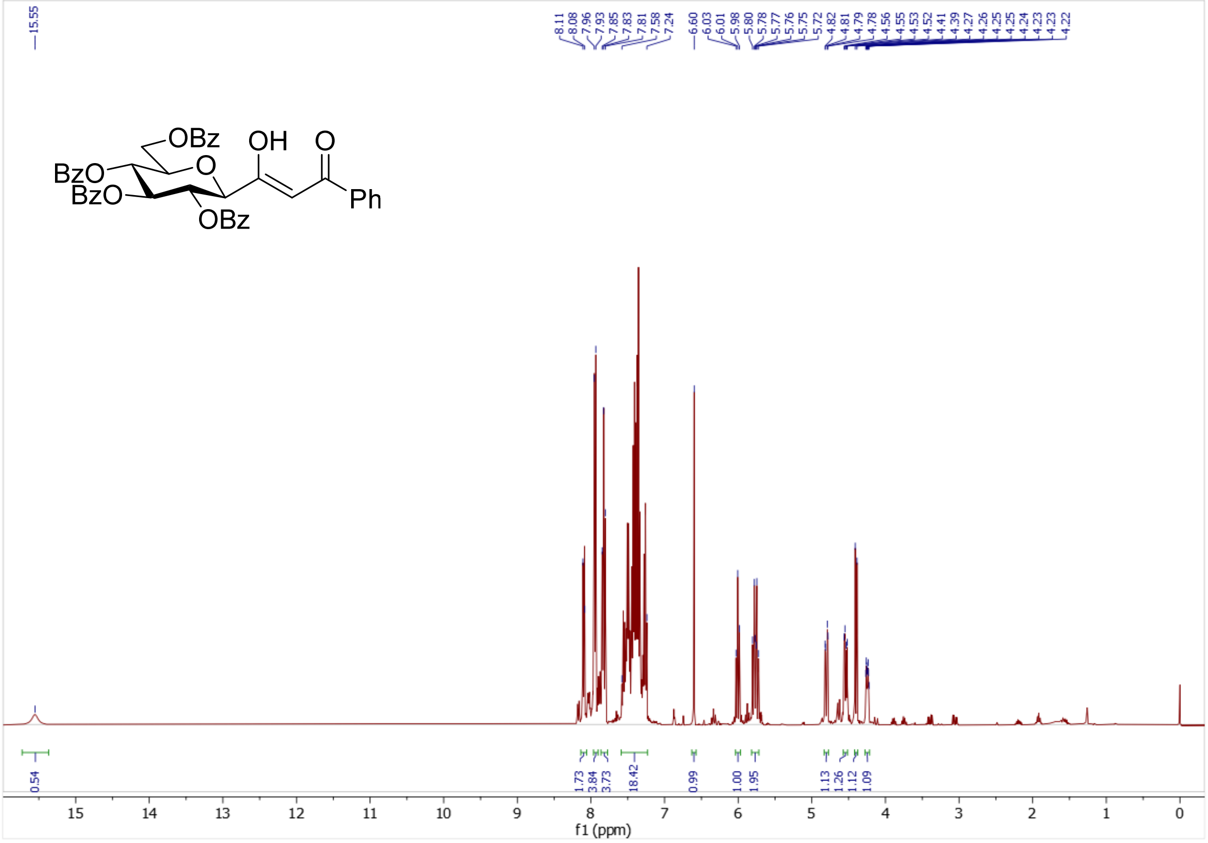

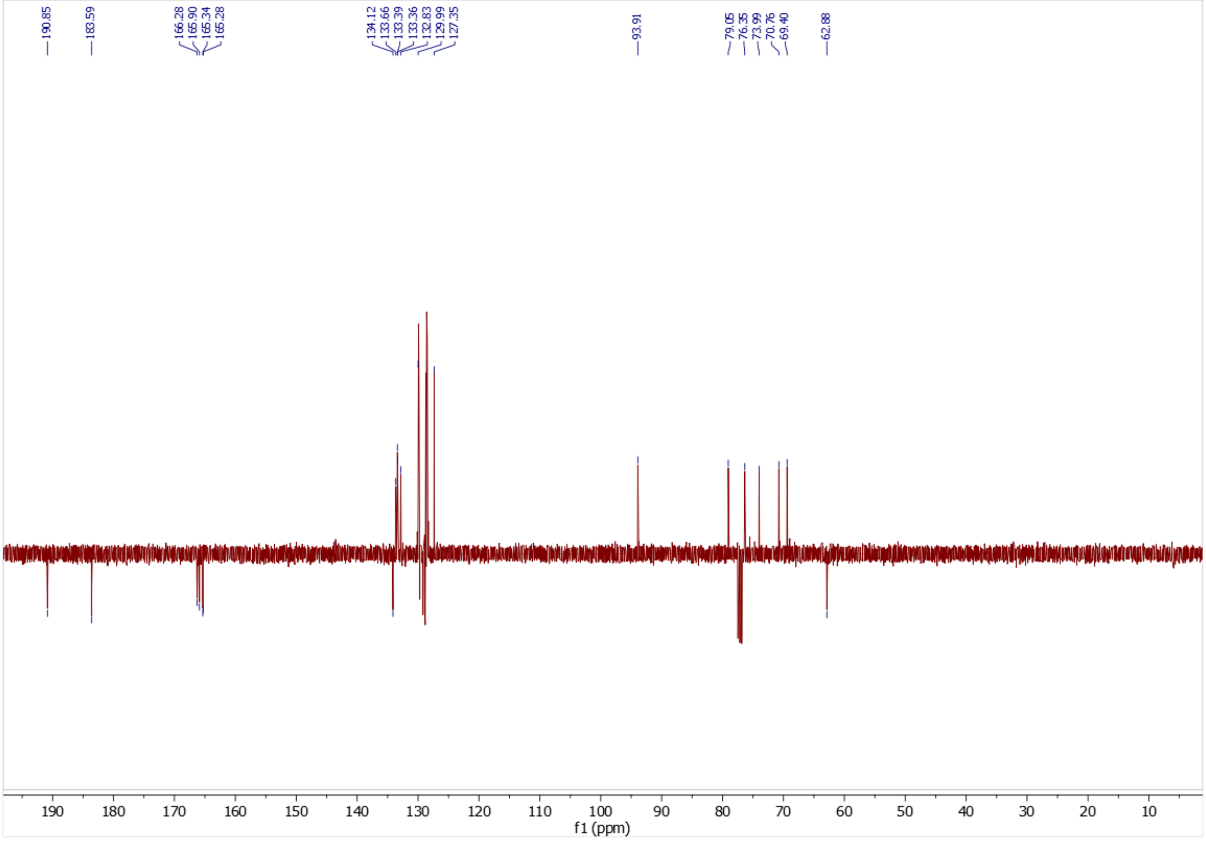

^{1}H (360 MHz) and ^{13}C J-MOD (90 MHz) NMR spectra of compound **8** in $CDCl_3$

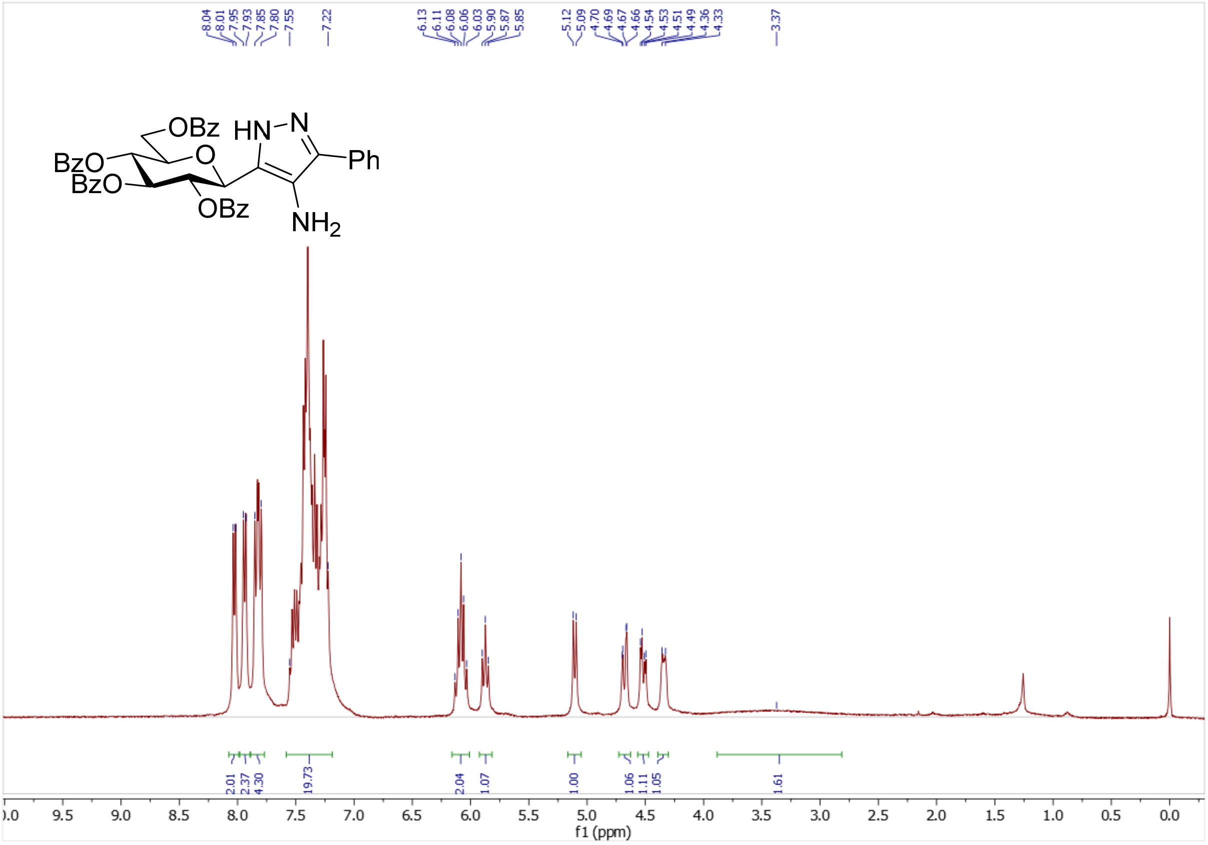

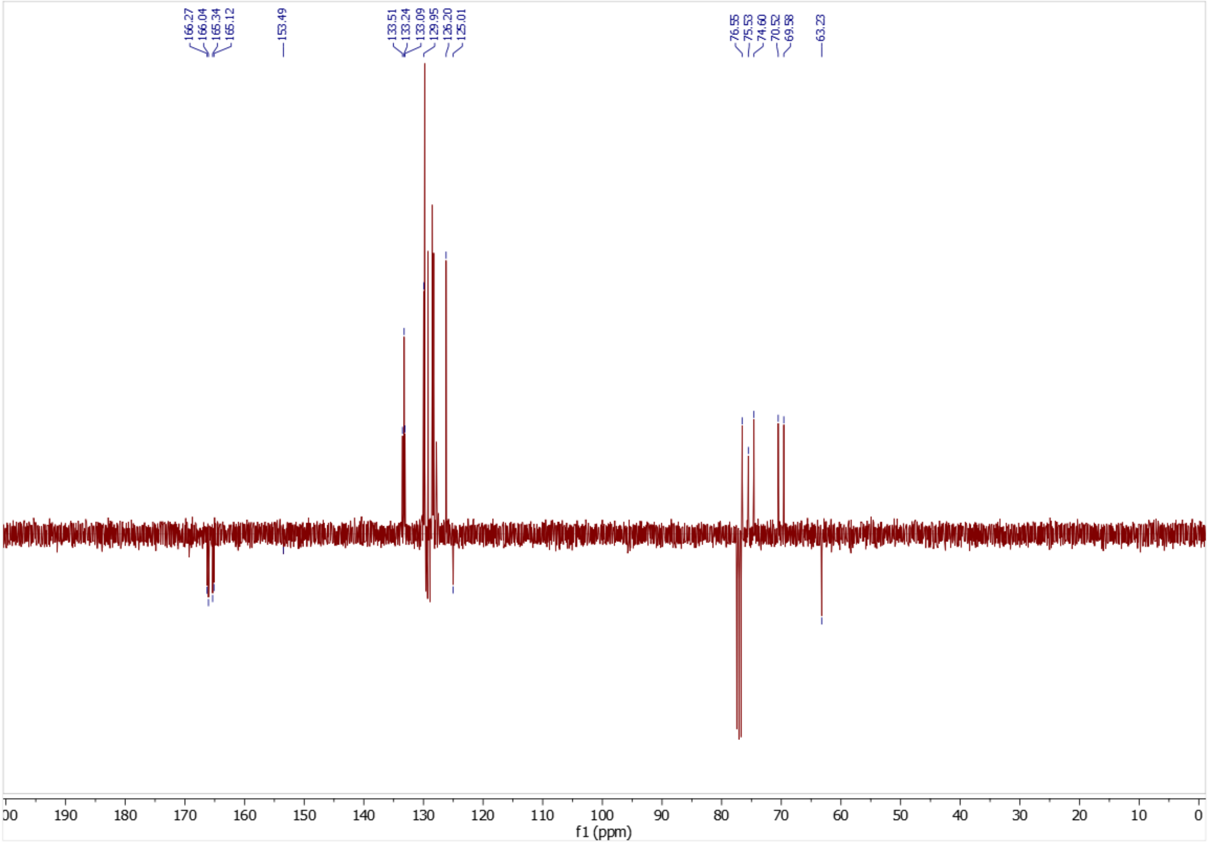

^{1}H (360 MHz) and ^{13}C J-MOD (90 MHz) NMR spectra of compound **9** in $CDCl_3$

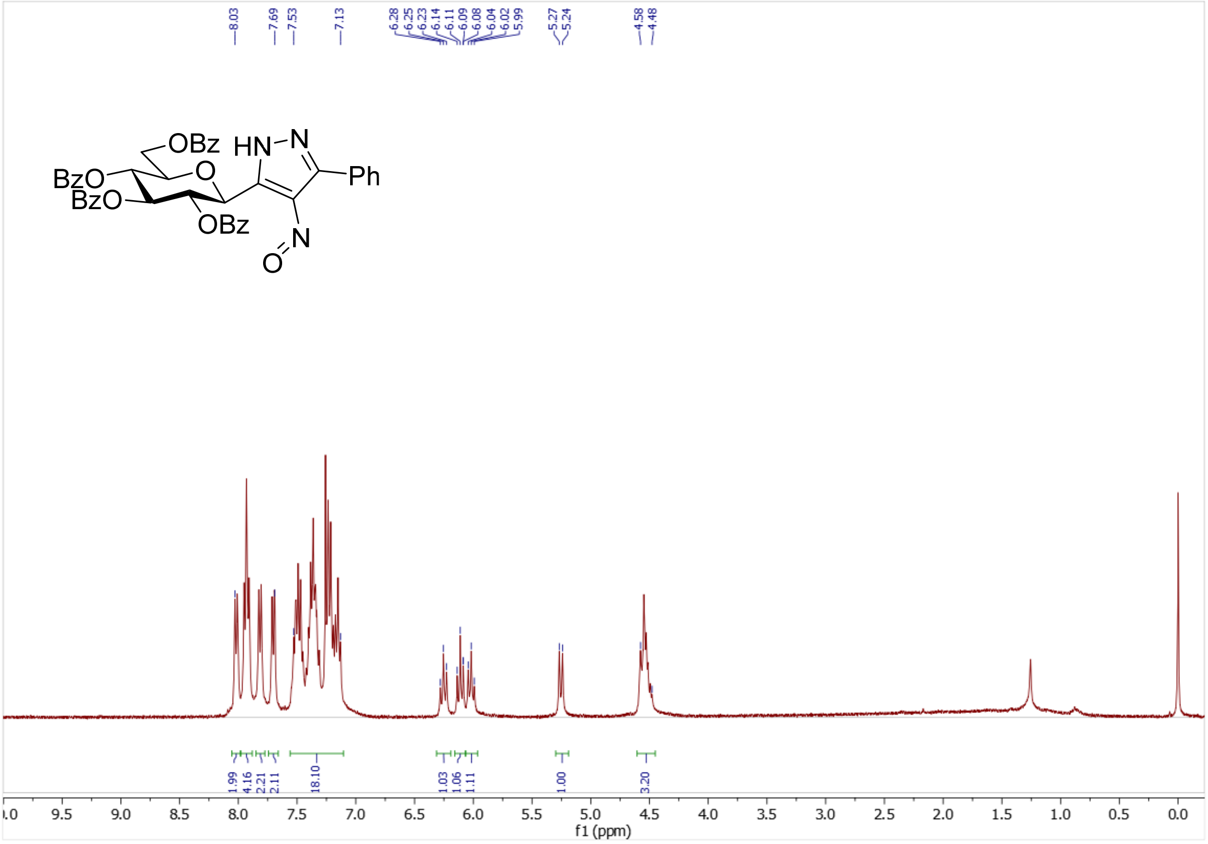

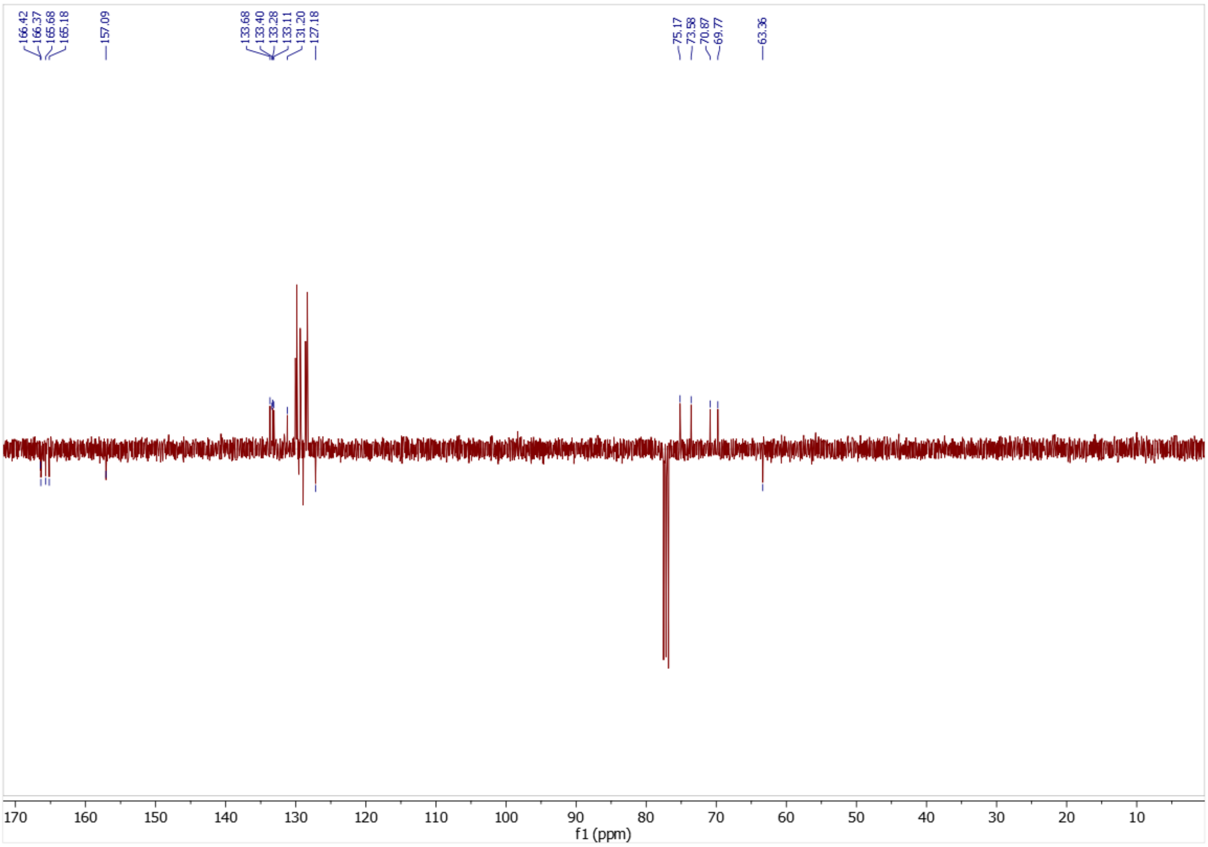

^{1}H (360 MHz) and ^{13}C J-MOD (90 MHz) NMR spectra of compound **10** in $CDCl_3$

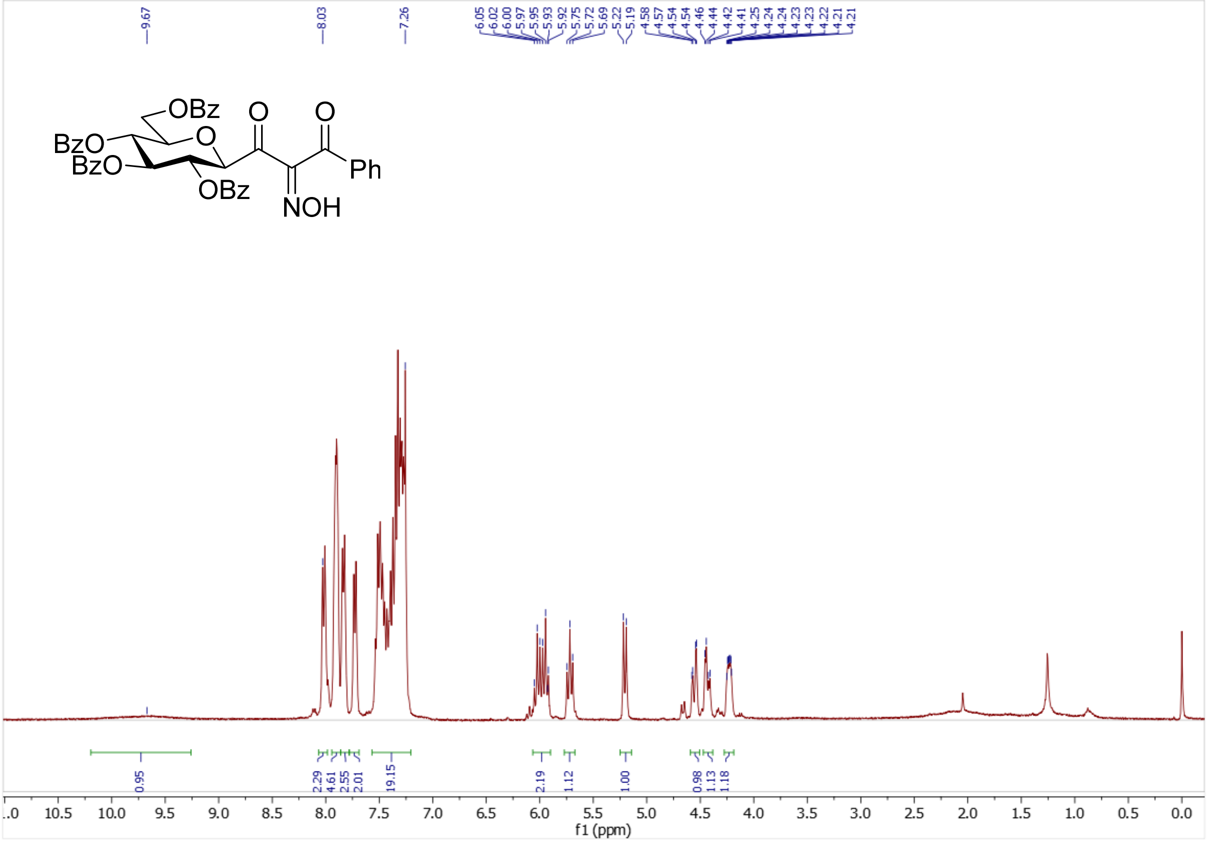

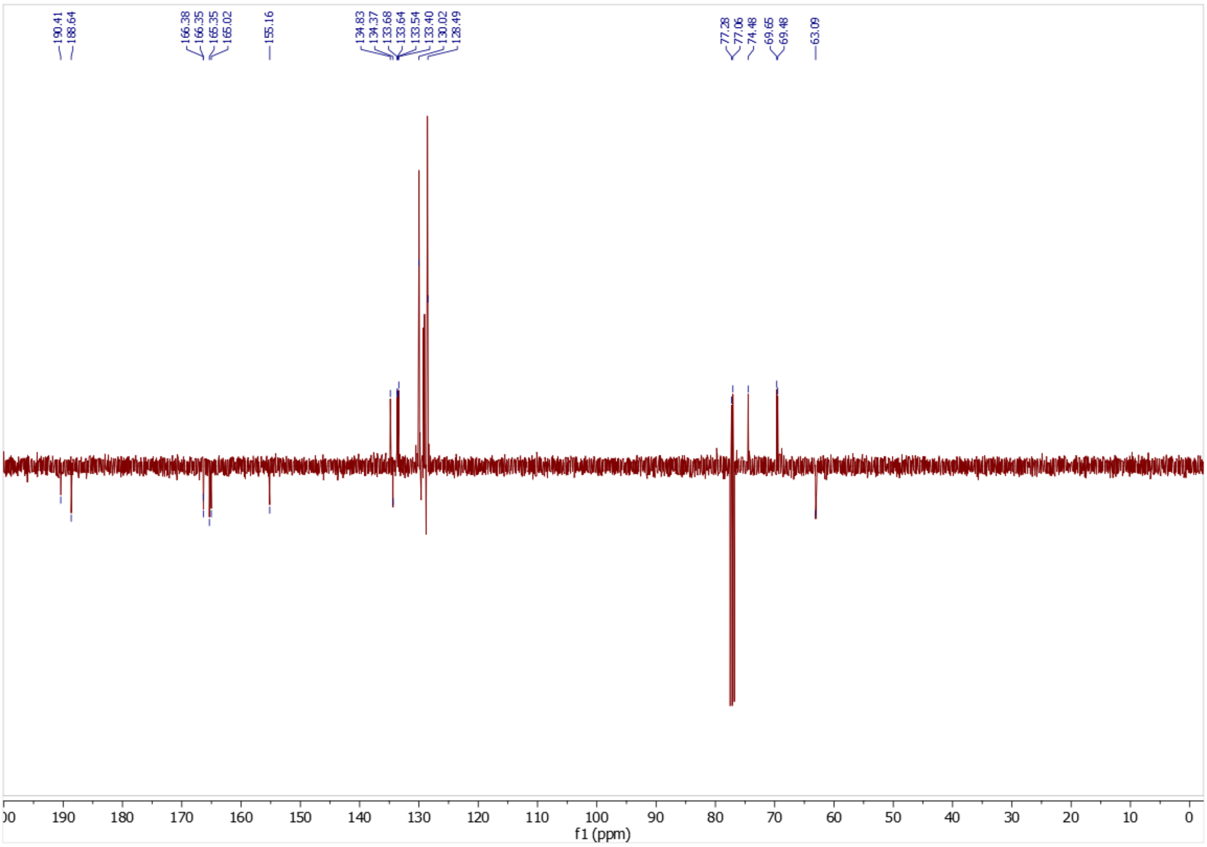

^{1}H (360 MHz) and ^{13}C J-MOD (90 MHz) NMR spectra of compound **11** in $CDCl_3$

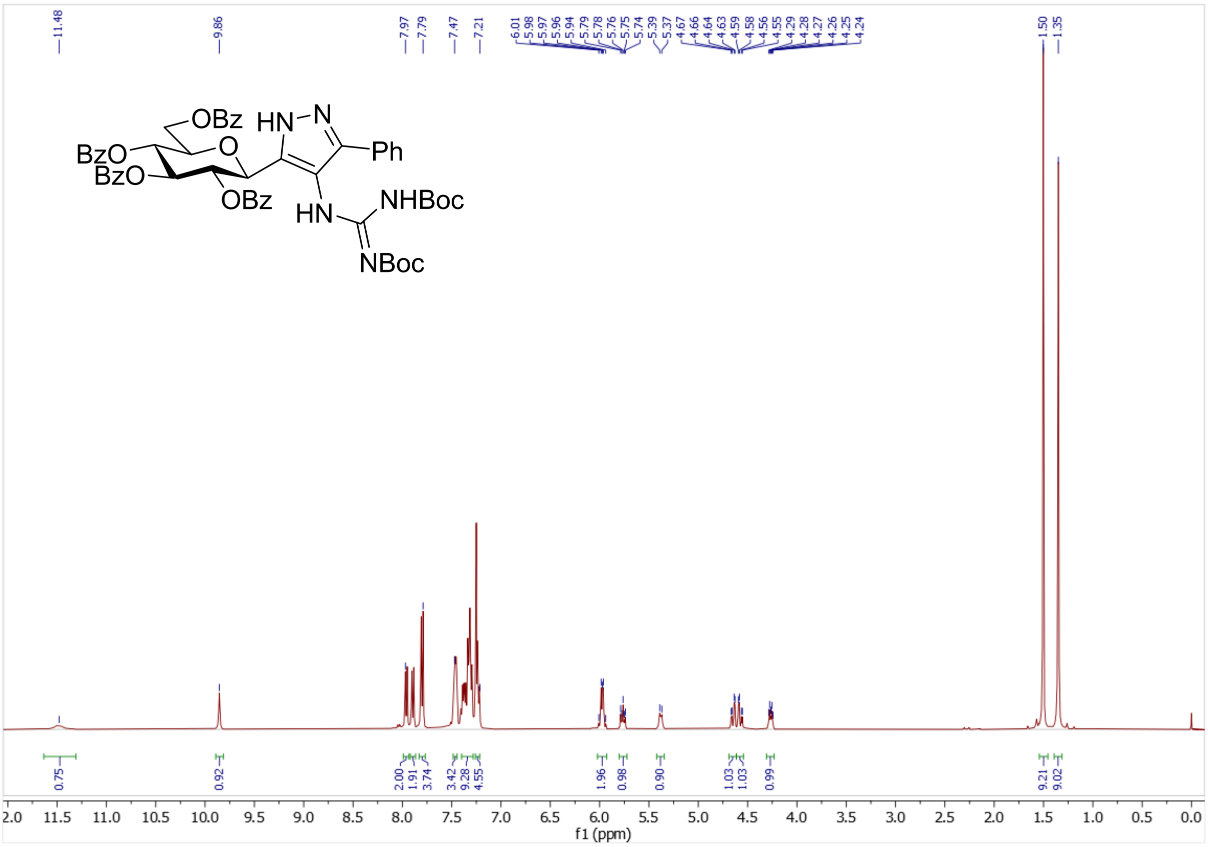

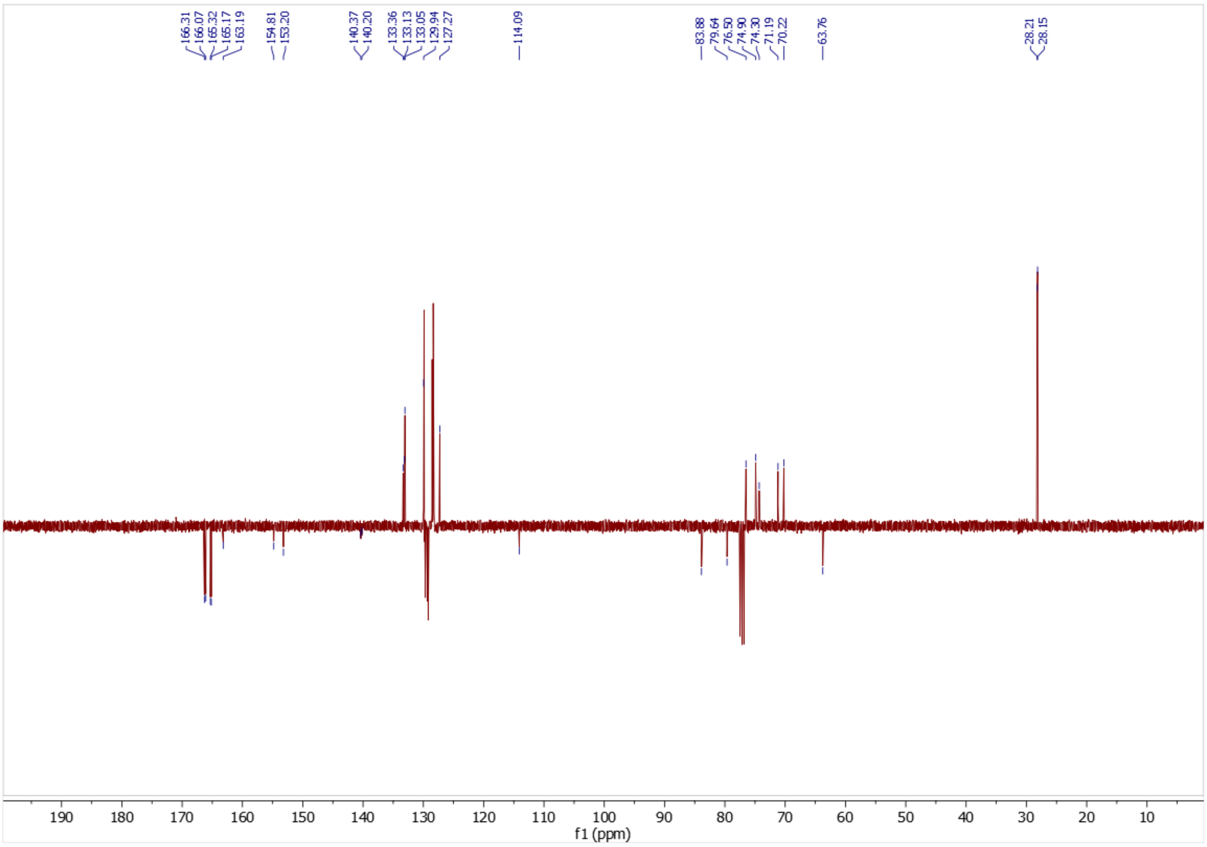

Supplement: Supplementary file 1 [file molecules-28-03005-s001.zip › molecules-2279059-supplementary.pdf]
